# Optimisation of peptides targeting reverse transcriptase HIV-1 using QSAR, machine learning, and computational approaches

**DOI:** 10.3389/fphar.2025.1707377

**Published:** 2025-12-10

**Authors:** Fachrur Rizal Mahendra, Indira Prakoso, Alfa Marzelino, Muhamad Rizqy Fadhillah, Syukriyansyah, Muhammad Marsha Azzami Hasibuan, Juniza Firdha Suparningtyas, Mikael Kristiadi, Aprijal Ghiyas Setiawan, Faris Izzatur Rahman, Anissa Nofita Sari, Nauval Rajwaa Raysendria, Ilham Kurniawan, Wawaimuli Arozal, Kusmardi Kusmardi

**Affiliations:** 1 Bioinformatics Research Center, Indonesian Institute of Bioinformatics (INBIO Indonesia), Malang, East Java, Indonesia; 2 Department of Biochemistry, Faculty of Mathematics and Natural Sciences, Bogor Agricultural University, Bogor, West Java, Indonesia; 3 Faculty of Medicine, Universitas Indonesia, Depok, West Java, Indonesia; 4 Department of Pharmacology and Therapeutic Faculty of Medicine, Universitas Indonesia, Depok, West Java, Indonesia; 5 Department of Information Systems, Faculty of Technology and Computer Science, Indraprasta PGRI University, Jakarta, Indonesia; 6 Pharmaceutical Research and Development Laboratory of FARMAKA TROPIS, Faculty of Pharmacy, Mulawarman University, Samarinda, East Kalimantan, Indonesia; 7 Biochemistry and Biomolecular Engineering Research Division, Faculty of Mathematics and Natural Sciences, Institut Teknologi Bandung, Bandung, West Java, Indonesia; 8 Indonesian Solidarity Genomics Laboratory (GSI-Lab), Jakarta, Indonesia; 9 Research Center for Vaccine and Drug, National Research and Innovation Agency (BRIN), Bogor, West Java, Indonesia; 10 Faculty of Biology, Universitas Gadjah Mada. Jl. Teknika Selatan, Yogyakarta, Indonesia; 11 Faculty of Medicine, Universitas Pembangunan Nasional Veteran Jawa Timur, Surabaya, Indonesia; 12 Anatomical Pathology, Faculty of Medicine, Universitas Indonesia, Depok, West Java, Indonesia; 13 Human Cancer Research Center, IMERI, Universitas Indonesia, Depok, West Java, Indonesia; 14 Drug Development Research Center, IMERI, Universitas Indonesia, Depok, West Java, Indonesia

**Keywords:** drug discovery, HIV, machine learning, peptide, QSAR, quantum mechanics, reverse transcriptase

## Abstract

The emergence of drug resistance and adverse side effects associated with current HIV-1 reverse transcriptase (RT) inhibitors underscores the need for novel therapeutic strategies. Peptide-based drugs offer high specificity and lower toxicity, but their development is challenged by the vast combinatorial space of possible sequences and formulation issues. This study aims to identify potent tripeptide-based inhibitors targeting HIV-1 RT through an integrated computational pipeline combining machine learning, QSAR modeling, and *in silico* validation techniques. From 2,197 screened tripeptides, three candidates, namely FHW, HFW, and HHW, emerged with superior predicted affinity, stability, and drug-like properties. Among them, FHW exhibited the strongest interaction with HIV-1 RT, with a binding energy of −63.50 kcal/mol using MM/GBSA, outperforming the reference drug Nevirapine. FHW peptide also shows four same residues with Nevirapine, including Leu100, Val106, Tyr181, and Tyr188 by hydrophobic contacts. DFT analysis revealed favorable electronic properties, including a low HOMO-LUMO gap (4.73 eV) and high electrophilicity index (13.60). Based on these findings, the FHW peptide demonstrates the highest electrophilicity index among the four ligands, indicating superior electrophilic character relative to Nevirapine. PerMM simulations further indicated consistently negative energy profiles for FHW during membrane translocation and reflecting favorable interactions with the lipid environment. Molecular dynamics simulations confirmed the structural stability of the FHW–RT complex over a 100 ns trajectory. Collectively, these findings identify FHW as the most promising tripeptide-based RT inhibitor with potential for development into next-generation HIV therapeutics, with HFW and HHW as alternative candidates.

## 1 Introduction

Human Immunodeficiency Virus (HIV) continues to pose a significant global health burden more than 4 decades after it first emerged as a pandemic. According to the Joint United Nations Programme on HIV/AIDS (UNAIDS, 2024), an estimated 39.9 million people were living with HIV globally in 2023. Despite substantial progress in antiretroviral therapy (ART), approximately 1.3 million individuals acquired new HIV infections in the same year. Two major classes of antiretroviral drugs, nucleoside reverse transcriptase inhibitors (NRTIs) and non-nucleoside reverse transcriptase inhibitors (NNRTIs), constitute the backbone of HIV treatment. These drugs act by targeting the HIV reverse transcriptase (HIV-RT), an RNA- and DNA-dependent DNA polymerase responsible for the reverse transcription of the viral RNA genome into DNA. Inhibiting HIV-RT is a critical therapeutic strategy, as it prevents the virus from converting its RNA into DNA, thereby disrupting its ability to integrate into the host genome and replicate. However, the clinical use of NRTIs and NNRTIs is often limited by a range of adverse effects, including pruritus, fatigue, nausea, myalgia, neuropathy and lactic acidosis, as well as the emergence of drug-resistant viral strains (Margolis et al., 2014; Shi et al., 2016). A study by de los Rios et al. (2021) revealed that 43.6% of individuals on ART experienced side effects, with 65.8% reporting gastrointestinal symptoms such as stomach upset, pain, and diarrhea. These adverse effects not only affect quality of life but also contribute to non-adherence, with many patients missing one or more doses of ART in the past month to avoid discomfort. The failures of several first-generation and second-generation small molecule drug-based anti-HIV therapies in various stages of clinical trials are an indication that there is a need for a paradigm shift in the future designs of anti-HIV therapeutics. The limitations and failures of several first- and second-generation small-molecule anti-HIV therapies in clinical trials underscore the urgent need for a paradigm shift in the design of future HIV therapy.

Peptide-based therapeutics have emerged as a promising alternative to small-molecule drugs which offer several notable advantages, including high specificity, potency, and reduced side effects (Fosgerau and Hoffmann, 2015). Composed of short amino acid sequences, peptides can interact with target proteins more effectively than small molecules due to their larger binding surfaces and the ability to form stronger and more specific interactions (Craik et al., 2013). This specificity contributes to a lower likelihood of off-target effects, reduced drug–drug interactions, and minimal toxicity, as peptides are typically metabolized into non-toxic amino acids (Olmez et al., 2012). Additionally, peptide therapeutics are generally less susceptible to the development of drug resistance. The high degree of structural specificity required for effective peptide binding means that viruses must undergo more substantial structural mutations to evade inhibition, making resistance less likely to emerge. Despite these advantages, peptide-based therapies have several limitations, such as poor *in vivo* stability and limited oral bioavailability, which complicate formulation and delivery (Choonara et al., 2014). Currently, two peptide-based drugs are approved for HIV treatment. The first is Enfuvirtide, a fusion inhibitor, which interferes with the conformational changes in the viral envelope protein gp41, thereby preventing membrane fusion. The second is Maraviroc, an entry inhibitor that blocks the interaction between gp120 and the CCR5 co-receptor, thereby preventing viral entry into host cells (Shi et al., 2016). However, to date, there are no approved peptide-based therapeutics targeting HIV-RT, highlighting a gap in therapeutic development and an opportunity for future research.

The design of peptide-based drugs presents significant challenges, primarily due to the immense combinatorial space of potential peptide sequences. For instance, a simple tripeptide constructed from the 20 standard amino acids results in 8,000 unique combinations which needs experimental screening that both time-consuming and cost-prohibitive. This complexity underscores the need for efficient computational strategies to prioritize peptide candidates with the highest therapeutic potential. Computational approaches such as Quantitative Structure–Activity Relationship (QSAR) modeling and machine learning (ML) have become important to modern peptide drug discovery. These methods enable rapid *in silico* prediction of bioactivity, facilitating the selection of promising candidates from large peptide libraries and significantly accelerating the drug development pipeline. QSAR modeling is a ligand-based approach that predicts biological activity by correlating structural features with functional outcomes. Early QSAR studies relied on simple physicochemical descriptors (1D-QSAR). However, modern QSAR models incorporate multidimensional molecular representations, such as 2D, 3D, and 4D descriptors, and are often combined with machine learning algorithms to improve predictive accuracy (Mao et al., 2021). These advanced models can be employed to estimate quantitative outcomes such as pIC_50_ values, or to classify compounds based on activity, thus providing valuable insights for rational peptide design.

This study presents potential candidates of novel peptide-based inhibitors toward HIV RT which offer an alternative to current antiretroviral drugs that often cause adverse side effects and contribute to drug resistance. By integrating ML techniques with QSAR modeling, we enhance the predictive accuracy of peptide-drug interactions while efficiently screening vast molecular libraries (Dogan and Durdagi, 2021; Baassi et al., 2023). Furthermore, *in silico* verification methods, including molecular docking simulations, were employed to evaluate the binding affinities and interaction patterns between the peptide candidates and the HIV RT active site. To refine the selection of top candidates, Molecular Mechanics-Generalized Born Surface Area (MM/GBSA) scoring was applied, providing a more robust assessment of binding free energies and stability. This multi-tiered computational approach not only accelerates the identification of promising drug candidates but also improves the reliability of virtual screening pipelines. The findings provide a foundation for future experimental validation with peptide candidates that can be evaluated *in vitro* and *in vivo* to assess their biological efficacy and pharmacological safety.

## 2 Materials and methods

**FIGURE 1:**
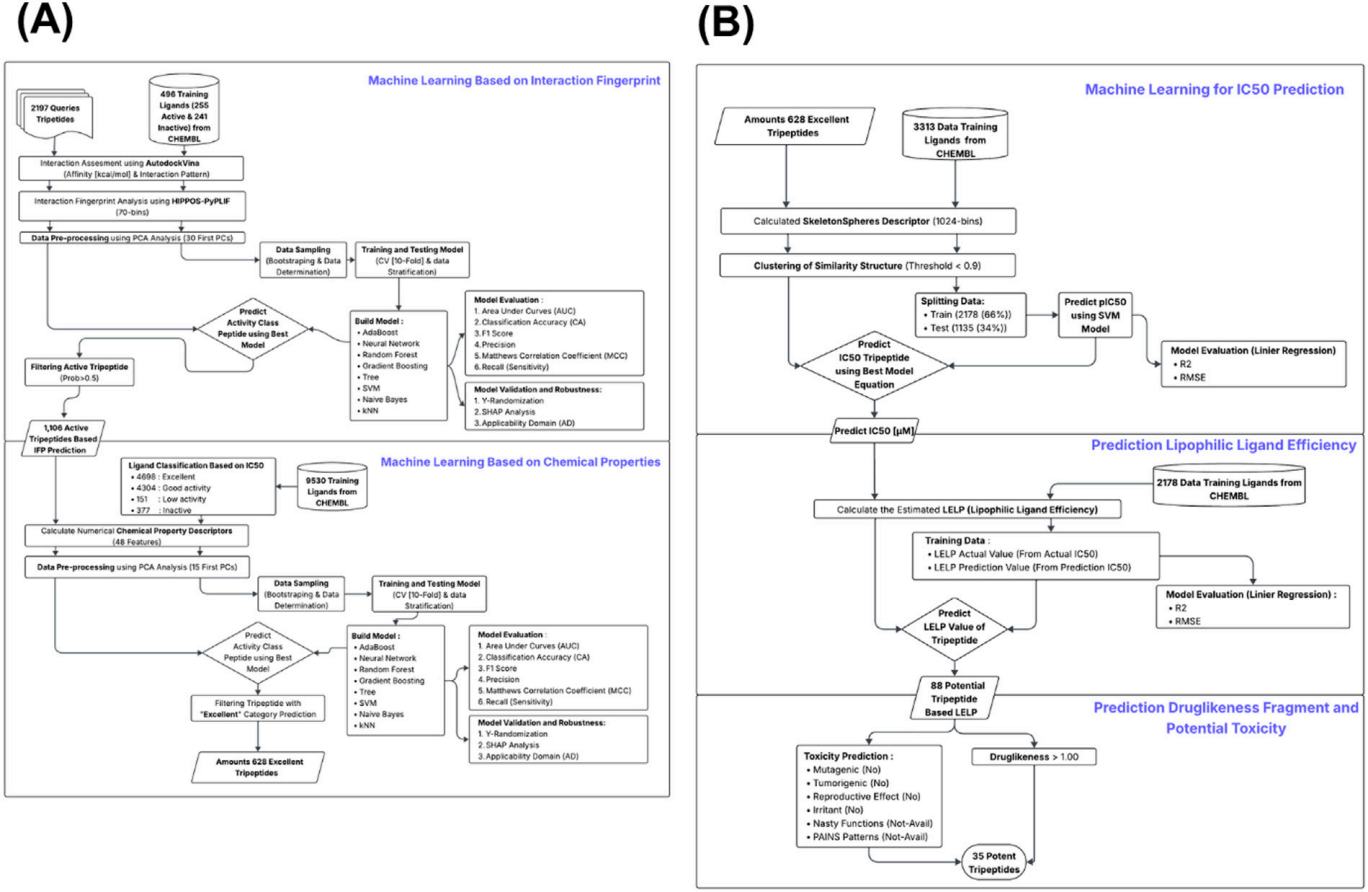
Schematic overview of the research workflow. **(A)** Machine learning–based categorical assessment integrating interaction fingerprint and molecular descriptor features. **(B)** Machine learning–based prediction of bioactivity (IC_50_), ligand efficiency–lipophilicity (LELP) score, and drug-likeness properties.

This study employed a multi-stage machine learning approach to identify potent tripeptides with high activity, efficiency, and low toxicity. A collection of tripeptides was evaluated using a set of ligand data databases, including datasets for interaction fingerprints, chemical descriptors, IC_50_ prediction, and lipophilic efficiency. Docking with AutoDock Vina and AutoDock4, followed by interaction fingerprint generation using HIPPOS-PyLIF, was designed for Interaction-based modeling. Reduced feature dimensions before training models with various algorithms (e.g., Support Vector Machine (SVM), random forest, neural networks) were done using Principal Component Analysis (PCA). Peptides with high predicted activity (probability >0.5) were selected. Separately, chemical descriptors were calculated and used to classify activity levels (excellent, good, low, inactive). Peptides predicted as “excellent” were prioritized. IC_50_ values were predicted using structural fingerprints based on the SVM regression algorithm, with performance evaluated *via R* ^2^ and RMSE. For lead optimization (predicted favorable ADMET properties), lipophilic-ligand efficiency (LELP) was estimated based on IC_50_ prediction (−10< LELP <10). Final peptide candidates were filtered based on drug-likeness (score >1.00) and absence of predicted toxicity. This workflow yielded potent tripeptides with strong predicted efficacy and favorable safety profiles (Figures 1A,B).

### 2.1 Data collection for machine learning workflows

The first step is the training data for interaction fingerprint (IFP) analysis were taken from the Directory of Useful Decoys-Enhanced (DUD-E) database (Mysinger et al., 2012), covering active ligands that are known experimentally to inhibit the HIV type 1 Reverse Transcriptase enzyme (HIV-1-RT), as well as decoy ligands that share physical-chemical similarities but differ significantly in topology and conformation, thereby assuming an inactive role. From DUD-E, a total of 496 ligands (255 active and 241 inactive) were obtained in various formats, such as Mol2, whose activity against RT-HIV is already known. The next is a total of 9,530 ligands associated with RT-HIV were also extracted from the ChEMBL for bioactivity prediction using IC_50_ (Gaulton et al., 2017) database integrated into DataWarrior V6.1.0. (Sander et al., 2015). The queries were converted the data contains IC_50_ values in Simplified Molecular-Input Line Entry System (SMILES) format. For 2,197 tripeptide query structures (excluding residues that do not contain simple nonpolar aliphatic side chains such as glycine, alanine, leucine, isoleucine, valine, and methionine, as well as residues with rigid side chains such as proline), were generated using PeptideConstructor 0.2.1 packages in Python 3.11.0 (Tien et al., 2013) and rdkit-pypi packages using 12 L-amino acid, excluding eight non-polar aliphatic R-groups.

#### 2.1.1 IFP prediction

The 3D IFP analysis between test ligand, the query tripeptide, and receptors using Python-based Protein–Ligand Interaction Fingerprinting (PyPLIF-HIPPOS) tools (Istyastono et al., 2020). The analysis was carried out using the output of the virtual screening Autodock Vina (Trott and Olson, 2010) in the previous step, based on the steps contained in Radifar et al. (2012). HIPPOS performs a similarity comparison between the test ligand and the reference ligand that is experimentally tethered to the PDB structure. The IFP calculation involved both training ligands from ChEMBL and query test tripeptides. The results obtained from the IFP analysis were binary data (70 bins) containing information on the interaction fingerprint patterns between ligands and the receptor, including interactions such as apolar, aromatic face-to-face, aromatic edge-to-face, hydrogen bonds (acceptor and donor), and electrostatic interactions (positive and negative).

#### 2.1.2 Bioactivity and LELP prediction using chemical descriptors

The information retrieved from chemical property descriptors using DataWarrior V6.4.2, which involves ligand as train and tripeptide as test queries, where initially the ligands train were classified based on the IC_50_ (µM) range into four classes: (UNAIDS, 2024): excellent (<1 µM), (Margolis et al., 2014), good activity (1–100 µM), (Shi et al., 2016), low activity (100–200 µM), and (de los Rios et al., 2021) inactive (>200 µM). The chemical property descriptors calculated include 48 numerical features, which contain druglikeness, atom counts, ring counts, functional groups, and 3D parameters (excluding categorical parameters). The LELP data is generated from IC_50_ from subsequent queries.

### 2.2 Machine learning test

#### 2.2.1 Machine learning model build

In this study, two machine learning approaches were applied to predict the activity of tripeptides as active ligands against RT-HIV as the protein target, based on IFP and chemical properties (See Figure 1). A machine learning model was built using Visual Code Studio (Python Version 11) and Orange software 3.37.0 (Demšar et al., 2013). A total of 2,196 tripeptide candidates were examined, and 496 training ligands (255 active and 241 inactive) were taken from the ChEMBL database. For the IFP-based approach, an initial evaluation was conducted using Autodock Vina to obtain the binding affinity data (kcal/mol) and interaction patterns, which were then analyzed using PyPLIF-HIPPOS and converted into 70 binary fingerprints. This data was then processed through PCA, considering the first 30 components. Next, the data was sampled using bootstrapping and data stratification, then used for model training and testing using cross-validation (10-fold CV). Eight algorithms were used to build the model, including AdaBoost, Artificial Neural Networks, Random Forest, Gradient Boosting, Decision Trees, SVM, Naïve Bayes, and k-NN. The models were evaluated based on Area Under the Curve (AUC), Classification Accuracy (CA), F1-score, precision, Matthews Correlation Coefficient (MCC), and sensitivity (recall). The best model was used to predict the activity class of tripeptides, and peptides with an active classification probability above 0.5 were filtered for further IFP-based prediction.

For the chemical property-based approach, 9,530 training ligands from ChEMBL were classified based on IC_50_ values into four classes: Excellent (4,698), Good activity (4,304), Low activity (151), and Inactive (377). All ligands were converted into 48 numerical chemical property descriptors. The data was then processed using PCA (first 15 principal components) and sampled as in the previous IFP approach. Model training and testing procedures, as well as performance evaluation, were performed using the same methods and algorithms. The best model was then used to predict the activity class of tripeptides based on their chemical properties, and peptides with the highest probability in the “Excellent” category were selected as priority candidates.

#### 2.2.2 Machine learning model assessment

To evaluate the statistical significance of the model and prevent random correlations, a Y-randomization test was performed using 100 random permutations of the response variable. For each permutation, the dependent variable (Y) was randomly shuffled while keeping the feature matrix (X) unchanged. The both models (IFP and chemical descriptor) was trained and evaluated using the same data split and evaluation metrics (accuracy, AUC, precision, recall, F1, and MCC). The statistical significance of the observed model was quantified using the following permutation-based p-value:

$$p=\frac{N_{perm}\left(score_{perm}\geq score_{orig}\right)+1}{N_{perm}+1}$$

Where *N*_*perm*_ denotes the number of random permutations. A low p-value (<0.05) indicates that the model performs significantly better than random chance

To enhance model transparency and component most responsible for the observed predictions, SHapley Additive ex Planations (SHAP) analysis was performed using the TreeExplainer algorithm (Lundberg et al., 2017). This approach quantifies the marginal contribution of each input feature to the model’s output based on cooperative game theory, enabling both global and local interpretability of predictions. Since the classification task involved multiclass outputs, SHAP values were computed for each class and then aggregated by calculating the mean absolute SHAP value across all classes to obtain the overall feature importance.

To ensure the reliability and generalizability of the developed machine learning model, the applicability domain (AD) was systematically evaluated using both leverage and Mahalanobis distance based metrics. This dual approach is widely recognized in QSAR studies to define the region of chemical space within which model predictions are considered reliable (Roy et al., 2015; Sahigara et al., 2012; Gramatica, 2007). The leverage (h_i_) of each compound was computed from the hat matrix as follows:

$$hi=x_{i}^{T}{\left(X^{T}X\right)}^{-1}x_{i}$$

where *xi* the descriptor vector of the *ith* compound, and X is the descriptor matrix of the training set.

A critical leverage value (h*) was defined as:

$$h*=\frac{3\left(p+1\right)}{n}$$

where *p* is the number of model descriptors, and *n* is the number of training compounds.

Compounds with hi > h* were considered outside the structural applicability region of the model.

To complement the leverage-based AD, Mahalanobis distance (MD) was also calculated for each test compound as:

$${MD}_{i}=\sqrt{{\left(x_{i}-\mu \right)}^{T}\;\Sigma^{-1}\left(x_{i}-\mu \right)}$$

Where μ is the mean descriptor vector of the training set, and Σ is the covariance matrix.

A compound was considered outside the AD if its Mahalanobis distance exceeded the empirical threshold:

$${\text{MD}}_{\text{threshold}}=\text{mean}\left(\text{MD}\right)+3\;x\text{ SD}\left(\text{MD}\right)$$

### 2.3 QSAR model

From 9,530 ligands associated with experiments on HIV-1 reverse transcriptase enzymes from DataWarrior V6.4.2, training and testing data were divided at a ratio of 20:80 to predict tripeptide properties based on IC_50_ values, LELP, drug similarity, and fragment toxicity potential. First, tripeptides in the “excellent” activity category were analyzed alongside 3,313 training ligand data from ChEMBL using SkeletonSpheres descriptors (1024-bin) to capture molecular structure backbond. Structural clustering was then performed based on similarity with a threshold of 0.9. The clustered data were then divided into training data (2,178; 66%) and test data (1,135; 34%), and their pIC_50_ values were predicted using a SVM model. Model evaluation was performed using linear regression with *R* ^2^ and RMSE metrics. The best model prediction results were then used to predict the IC_50_ values of the candidate tripeptides. Based on this IC_50_ value, the actual (training data) and predicted (test data) LELP can be calculated based on (Hopkins et al., 2014) using the following equation:

$$LELP=\frac{LogP}{LE}$$

$$LE=1.37\;x\;(\frac{-\log_{10}\;\left({IC}_{50}\left(µM\right)\right)}{N_{Heavy\;atoms}}$$

The LELP model was evaluated using a linear regression model involving actual and predicted LELP values for 2,178 ligands from ChEMBL, which was then used to train a model that predicts LELP values for candidate tripeptides. This model was evaluated using *R* ^2^ and RMSE.

### 2.4 Drug-likeness

The drug-likeness criteria were determined based on predicted values of >1.00 using DataWarrior V6.4.2, according to (Dalafave, 2010; Sander et al., 2015). The drug-likeness of the compounds was assessed using, fragment-based approach derived from a database of ∼3,300 marketed drugs and ∼15,000 non-drug-like compounds (Fluka). The method calculates a drug-likeness score based on the presence of ∼5,300 distinct substructural fragments, each associated with a predefined score obtained from the logarithmic ratio of their occurrence in drug *versus* non-drug collections. The final drug-likeness value for each compound is computed as the sum of the individual fragment scores present in the molecule. Positive scores indicate the predominance of drug-associated fragments, whereas negative values reflect similarity to non-drug-like fragments. Compounds with positive drug-likeness values were considered favorable and prioritized for further pharmacokinetic and toxicity evaluation.

### 2.5 *In silico* verification

#### 2.5.1 Molecular docking assessment

Molecular docking assessment was conducted using multiple scoring from AutoDock Vina (AutoDock Vina v1.1.2 Linux x86) and AutoDock4 (AutoDock-GPU v1.6). Different docking tools was used to validate the docking of 35 tripeptide ligands to the Nevirapine binding site of the HIV-1 reverse transcriptase structure (PDB code: 3QIP) (Santos-Martins et al., 2021; Trott and Olson, 2010). The receptor was prepared and cleaned of missing atoms and inappropriate structures using PyMOL (TM) v3.1.6.1 and pdbfixer (https://github.com/openmm/pdbfixer) (GitHub - openmm/pdbfixer). The screening process began with the determination of the grid box size based on the lowest Root Mean Square Deviation (RMSD) values obtained from 50 re-docking simulations using both tools. In AutoDock Vina, the grid box was centered at X = 11.074, Y = 13.791, and Z = 17.342, with dimensions of 35 × 35 × 35 Å. This configuration yielded the lowest RMSD value of 0.118 Å and a docking affinity energy of −11.5 kcal·mol^−1^. In contrast, in AutoDock4, the grid box was also centered at X = 11.074, Y = 13.791, and Z = 17.342, but with dimensions of 60 × 60 × 60 Å, resulting in the lowest RMSD value of 0.85 Å and a docking affinity energy of −9.08 kcal mol^−1^.

For ligand preparation in AutoDock Vina, the 35 peptide ligands in PDB format were converted to PDBQT format using Open Babel v3.1.0 *via* the terminal. In contrast, for AutoDock4, ligand conversion to PDBQT format was performed using the ADFRsuite v1.0 program and the Grid Parameter File (GPF) for each ligand was generated using MGLTools v1.5.7 and executed with AutoGrid4 (Morris et al., 2009). AutoDock Vina employs a global search optimization strategy, with the default parameters set as follows: exhaustiveness = 8, number of output poses = 20, and minimum RMSD difference between poses = 1.00 Å. In contrast, AutoDock4 utilizes the Lamarckian Genetic Algorithm (LGA) by default, with both the number of LGA runs and the population size set to 100. For each ligand, the best docking pose along with its corresponding binding affinity was selected as the output. The RMSD of each docked ligand relative to its initial pose was calculated using RDKit v2025.3.3 (Sarthak Pa).

#### 2.5.2 MM/GBSA scoring

The MM/GBSA approach was applied to refine and validate the docking results by providing a more accurate estimation of binding free energy that accounts for solvation and entropic effects, which are often simplified or omitted in standard docking scoring functions. Compared to AutoDock4 and AutoDock Vina, MM/GBSA offers improved energetic realism and can better distinguish true binders from false positives. Validation of binding energy on ligand and receptor binding was conducted using MM/GBSA analysis based on the method of Uni-GBSA pipeline (Yang et al., 2023). Binding energy validation was carried out using the output results of *.pdb receptor and *.sdf ligand files (after conversion from pdbqt using open babel packages) in Autodock4 GPU screening. Then, the analysis was carried out using the unigbsa-pipeline tool on the Bash Script Linux.

#### 2.5.3 Visualization of interactions

The ligand–protein complex obtained from molecular docking was first converted from PDBQT to PDB format. The protein molecule was separated from the ligand and saved again in PDB format. The isolated ligand was then converted into SDF format. Both the protein and ligand structures were uploaded separately to the ProteinPlus web server for 2D interaction analysis. The PoseEdit tool within the server was used to visualize and analyze the two-dimensional interactions between the ligand and protein. To visualize the three-dimensional form, the docked ligand–protein complex was loaded into PyMOL. Surface depth analysis was performed to assess the spatial orientation and binding depth of the ligand within the protein’s binding pocket.

#### 2.5.4 Pocket assessment similarity

Pocket similarity analysis was performed using ProteinPlus web server (https://proteins.plus/) (Schöning-Stierand et al., 2022). The analysis used input files consisting of the protein structure in PDB format and the ligand structure in SDF format. The cavity pocket parameters evaluated included the number of hydrogen bond acceptors and donors, pocket depth (Å), hydrophobicity, presence of metal ions, number of protein heavy atoms, surface area (Å^2^), surface-to-volume ratio, and pocket volume (Å^3^).

#### 2.5.5 Quantum mechanical analysis

Quantum chemical calculations based on (Kohn and Sham, 1965; Cao et al., 2024) were performed to determine the energies of the Highest Occupied Molecular Orbital (HOMO) and the Lowest Unoccupied Molecular Orbital (LUMO) using the Density Functional Theory (DFT) method within the Jaguar module of the Schrödinger suite 2024-4. This process employed the Becke, 3-parameter, Lee-Yang-Parr (B3LYP)/6‒31 G basis set. The results were further refined with the Becke, 3-parameter, Lee-Yang-Parr dispersion correction (B3LYP-D3)3/6-311G basis set. The calculated ELUMO and EHOMO values were then used to determine various quantum chemical properties, including the HOMO-LUMO Gap (ΔG), chemical softness and hardness, global electrophilicity index, and electronegativity.

#### 2.5.6 Permeability prediction

Permeability of the peptide through biological membranes was analyzed using Permeability of Molecules across Membranes (PerMM) to calculate the energy profiles (Lomize et al., 2019). Meanwhile, membrane-binding affinities were analyzed using the Black Lipid Membrane (BLM) model.

#### 2.5.7 All atomic molecular dynamic simulation

Molecular dynamics (MD) preparations were performed by using CHARMM-GUI (Jo et al., 2008), and the simulation was performed through GROMACS (Abraham et al., 2015). In CHARMM-GUI, the solution builder feature was used for simulation preparation, including molecule preparation, ligand and ion handling, water box/solvent system, ion placement, and system assembly (Brooks et al., 2009). The parameters used are temperature 310K, NaCl 0.15% concentration, AMBER force field, and time 50 ns. The output of CHARMM-GUI is the initial topology file, along with the preparation for molecular dynamics simulation. Through GROMACS, minimization, equilibration, and simulation stages were carried out to see the trajectory and dynamics of each atom in the protein-ligand system. Visualisation was done using XMGrace tools (Osideko et al., 2025) to see the plot of root mean square deviation (RMSD), root mean square fluctuation (RMSF), and Radius of Gyration (Rg).

#### 2.5.8 Principal component analysis (PCA)

PCA was conducted using R-studio software, specifically employing the “mktrj.pca” function from the Bio3D package to analyze MD trajectories (Skjærven et al., 2014). The PCA calculation of molecular dynamics was performed using the trajectory (xtc) that had been cleaned from water molecules along with the protein structure file (pdb). PCA functioned as a multivariate exploratory tool to assess the distribution of internal energy variations within MD simulation structures, thereby facilitating the prediction of the system’s thermodynamic stability (David and Jacobs, 2014). Additionally, eigenvalue and eigenvector computations were performed for each complex to identify the two principal components with the greatest variance (Hess, 2002). The PCA model can be mathematically expressed as:

$$M=T_{K}P_{K}^{T}+R$$

where M is the resulting matrix product, $T_{K}$ represents sample correlations, $P_{K}$ corresponds to variable correlations, K is the number of parameters in the input, and R denotes the residual matrix. Beyond thermodynamic analysis, PCA was also utilized to investigate the directional movements of protein structures by measuring fluctuations in the Cα atoms within protein complexes (David and Jacobs, 2014). These atomic motions provide insights into the protein’s stability and functional dynamics. The PCA of Cα fluctuations begins by removing all solvent molecules and ions from the system, followed by calculating eigenvalues and eigenvectors for the top three principal components (PC1, PC2, and PC3), where these PCs represent vector values within the matrix (Gholam et al., 2024; Ezaj et al., 2022). The principal component values are derived from the covariance matrix using the formula:

$$C=VAV^{T}$$

Here, C is the covariance matrix, V is the matrix of eigenvectors, and A is the diagonal matrix of eigenvalues.

#### 2.5.9 Dynamic cross-correlation matrix (DCCM)

DCCM analysis was performed using R-studio with the “dccm” function in the Bio3D package (Grant et al., 2006). The DCCM plot is used to determine the correlation between the position of the Cα atom and other atoms in the structure, both directly and indirectly, especially the displacement that occurs in the Cα atom. The DCCM value is calculated based on the following mathematical equation:

$${DCCM}_{ij}=\frac{\langle \vec{d}i\;.\;\vec{d}j\rangle }{\sqrt{\langle {di}^{2}\rangle \langle {dj}^{2}\rangle }}$$

The values in and dj represent the shift from the initial position to the average position. The DCCMij value ranges from −1 to 1, where positive correlation indicates parallel interaction between atoms, while negative correlation describes antiparallel interaction between Cα atoms and other atoms in the protein structure.

#### 2.5.10 Free energy landscape (FEL)

Free energy landscape analysis was performed by evaluating the results of Gromacs simulations through fitted trajectory files (xtc), structure topology (tpr), and index files (ndx) (Yekeen et al., 2023). Throughout the entire trajectory interval (50 ns), the gmx covar command was run to generate output in the form of an eigenvalue file (eigenval.xvg) and an eigenvector file (eigenvec.trr), which represent the covariance of the overall atomic motion in the system. Next, gmx anaeig was used to project the first two principal components (PC1 and PC2), with the output being a PCA_2dproj.xvg file that describes the conformation distribution in PCA space. The projection results are then analyzed using gmx sham to construct a Gibbs free energy map (kJ/mol) in xpm format, which is then converted into images (eps and pdf) *via* gmx xpm2ps and ImageMagick. The Gibbs free energy calculation formula is as follows:

$${\Delta G}_{i}=-kT\left(\frac{P_{i}\left(r\right)}{P_{\max}\left(r\right)}\right)$$

In this equation, *k* represents the Boltzmann constant, while *T* denotes the simulation temperature. The value of *P*_*i*_(*r*) describes the probability of the system being in a particular state *i* determined by the reaction coordinate *r* (the observed parameter), and is obtained through a histogram of MD results. Meanwhile, *Pmax*(*r*) is the probability in the bin with the highest population or the most likely state. The change in free energy for state *i* is then expressed as Δ*G*_*i*_.

#### 2.5.11 Steered molecular dynamic simulation

Steered Molecular Dynamics (SMD) simulations were performed using CHAPERONg interfaced with GROMACS (Yekeen et al., 2023; Lemkul, 2019). The simulation was initiated by preparing the simulation parameters in the paraFile.par, which included the following settings: the protein structure (pdb format) was placed in a cubic simulation box and solvated with the TIP3P water model. Counterions (Na^+^ and Cl^−−^) were added to neutralize the system at a physiological salt concentration of 0.1 M, and the temperature was maintained at 310 K. System conditioning and atomistic parameters were defined using the CHARMM36 force field version July 2022 (http://mackerell.umaryland.edu/), In addition, using python script cgenff_charmm2gmx_py*_nx*.py from the same website are used to convert small molecule ligand file (str and mol2) topology parameters from CHARMM General Force Field (CGenFF) (https://app.cgenff.com/homepage) into a format that can be used by GROMACS. All simulations were carried out in fully automated mode, employing 2 CPU threads with non-bonded interactions calculated on the CPU. The system trajectory was processed in centered mode to remove translational artifacts, and 250 frames (400 ps) with timestep 2 fs were extracted to generate a representative molecular movie. Furthermore, molecular dynamics parameter (MDP) files were prepared for various steps, including ionization, energy minimization, equilibration (NPT), ligand pulling simulation, and the production run. Including the setup of the pulling coordinate velocity at 0.01 nm·ps^−1^, the pulling simulation with a harmonic potential force constant of 1,000 kJ ·mol^−1^·nm^−2^, the V-rescale thermostat (310 K), pressure control with the isotropic Parrinello–Rahman barostat (1 bar), and the use of Particle Mesh Ewald (PME) (grid spacing 0.12 nm, order 4).

#### 2.5.12 Pharmacokinetics prediction

Pharmacokinetic prediction of compounds was performed using the pkCSM platform (http://biosig.unimelb.edu.au/pkcsm/) (Pires et al., 2015), a graph-based signatures web server used to analyze ADME (Absorption, Distribution, Metabolism, and Excretion) properties of small molecules based on input in the form of compound structure representations in SMILES format. Each compound was transformed into SMILES notation and entered into pkCSM to obtain pharmacokinetic parameters. These prediction results were used to evaluate the potential of potential tripeptides.

#### 2.5.13 Toxicology prediction

The potential toxicity profile was assessed using DataWarrior V6.4.2 (Sander et al., 2015), which included parameters such as mutagenicity, tumorigenicity, reproductive effects, irritation, and the presence of harmful functions (nasty functional groups) or structural patterns such as Pan-Assay Interference Compounds (PAINS). Only compounds yielding results of “not available” or “not risky” were considered acceptable for further evaluation. In addition, toxicity endpoints were further evaluated using the pkCSM platform (http://biosig.unimelb.edu.au/pkcsm/) (Pires et al., 2015), including AMES mutagenicity, maximum tolerated dose (human) (MTD), hERG I and hERG II inhibition, oral rat acute toxicity (LD_50_), oral rat chronic toxicity (LOAEL), and hepatotoxicity.

## 3 Results

### 3.1 Machine learning model evaluation and external data prediction

**FIGURE 2:**
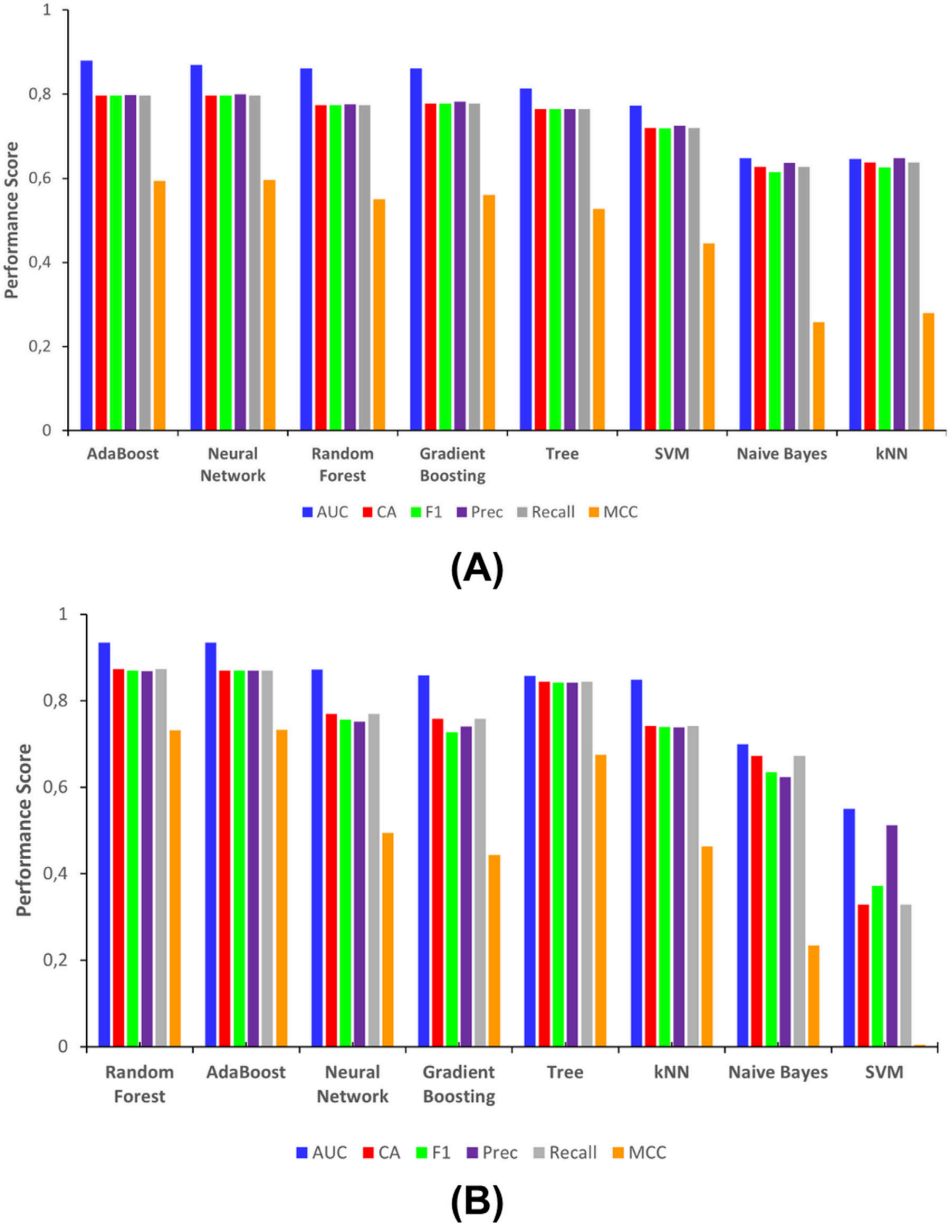
Evaluation of machine learning model based on **(A)** interaction fingerprints, and **(B)** chemical properties.

The classification performance of eight ML algorithms was evaluated using six metrics: AUC, CA, F1-score, Precision (Prec), Recall, and MCC (Supplementary Figures S1 and S2). The ensuing results are illustrated in Figure 2, with Figure 2A representing the first-layer classification based on the top 30 principal components (PCs) from interaction fingerprint features, and Figure 2B representing the second-layer classification based on the top 15 PCs from chemical property descriptors.

Among all models tested for IFP prediction (Figure 2A), AdaBoost, Artificial Neural Networks, and Random Forests consistently showed superior performance across all metrics, particularly in AUC values approaching or exceeding 0.9, indicating high discriminative power, as well as F1 scores (approaching 0.8) compared to other models, signifying a harmonic mean between precision and recall. Among these models, AdaBoost recorded the highest MCC value, indicating strong agreement between predictions and actual class labels. Conversely, the SVM, Naïve Bayes, and k-Nearest Neighbors (kNN) models demonstrated comparatively lower performance. Notably, Naïve Bayes and k-Nearest Neighbors (kNN) performed relatively poorly, particularly in terms of F1 and MCC metrics. The Decision Tree demonstrated intermediate performance, exhibiting stable yet unremarkable scores across all metrics. According to the IFP model prediction, out of 1,106 tripeptides from 2,197 were predicted to be active using the AdaBoost algorithm with minimum probability >0.5.

In the second layer model (Figure 2B), which uses chemical descriptor features, the overall classification performance is slightly better than in the first layer. The performance of eight machine learning models used to predict IC_50_ activity classification was also evaluated. It shows Random Forest, AdaBoost, and Neural Network models consistently outperformed others across most evaluation metrics, particularly in terms of AUC and F1-score, suggesting their robustness in handling multi-class classification tasks, maintaining their status as the top performers. Random Forest achieved the highest AUC values supported by its high MCC values, indicating its superior ability to distinguish between the different activity classes. Neural networks exhibited consistent performance, though they demonstrated a slight decrease for almost every single evaluation metric. Still, there was also a marked improvement in the AUC metric in comparison to interaction-based models. As in the initial layer, kNN, Naïve Bayes, and SVM demonstrated the least robust results, particularly in terms of MCC scores. Naïve Bayes and SVM showed comparatively lower performance, especially in MCC and F1, suggesting limitations in accurately capturing complex chemical patterns. Using chemical descriptor model prediction, among 1,106 peptides, 628 peptides have predicted excellent bioactivity with Random Forest approach with minimum probability >0.3.

#### 3.1.1 Model robustness and validation

The robustness and predictive reliability of the developed two-layer machine learning framework were comprehensively evaluated using standard performance metrics and Y-randomization testing to verify that the observed predictive power did not arise from chance correlations. In the first-layer model, based on IFP features and constructed using the AdaBoost algorithm, the classifier demonstrated excellent predictive performance with an AUC of 0.9348, classification accuracy (CA) of 0.8732, precision of 0.8693, recall of 0.8732, F1-score of 0.8700, and MCC of 0.7317 (Table 1).

**TABLE 1:** Performance metrics of the first-layer AdaBoost model based on IFP features.

Metrics	Value
Area under the ROC curve (AUC)	0.9348
Classification accuracy (CA)	0.8732
Precision	0.8693
Recall (sensitivity)	0.8732
F1-score	0.8700
Matthews correlation coefficient (MCC)	0.7317

To confirm that this performance was not the result of random associations, a Y-randomization (permutation) test with 100 iterations was conducted using AUC as the primary evaluation metric.

The randomized models yielded an average AUC of 0.50 ± 0.02, whereas the original AdaBoost model retained a substantially higher AUC of 0.9348, resulting in a permutation-based *p*-value of 0.0099 (p < 0.05). These results indicate that the AdaBoost classifier captures a genuine and statistically significant relationship between IFP-derived molecular patterns and peptide bioactivity.

**FIGURE 3:**
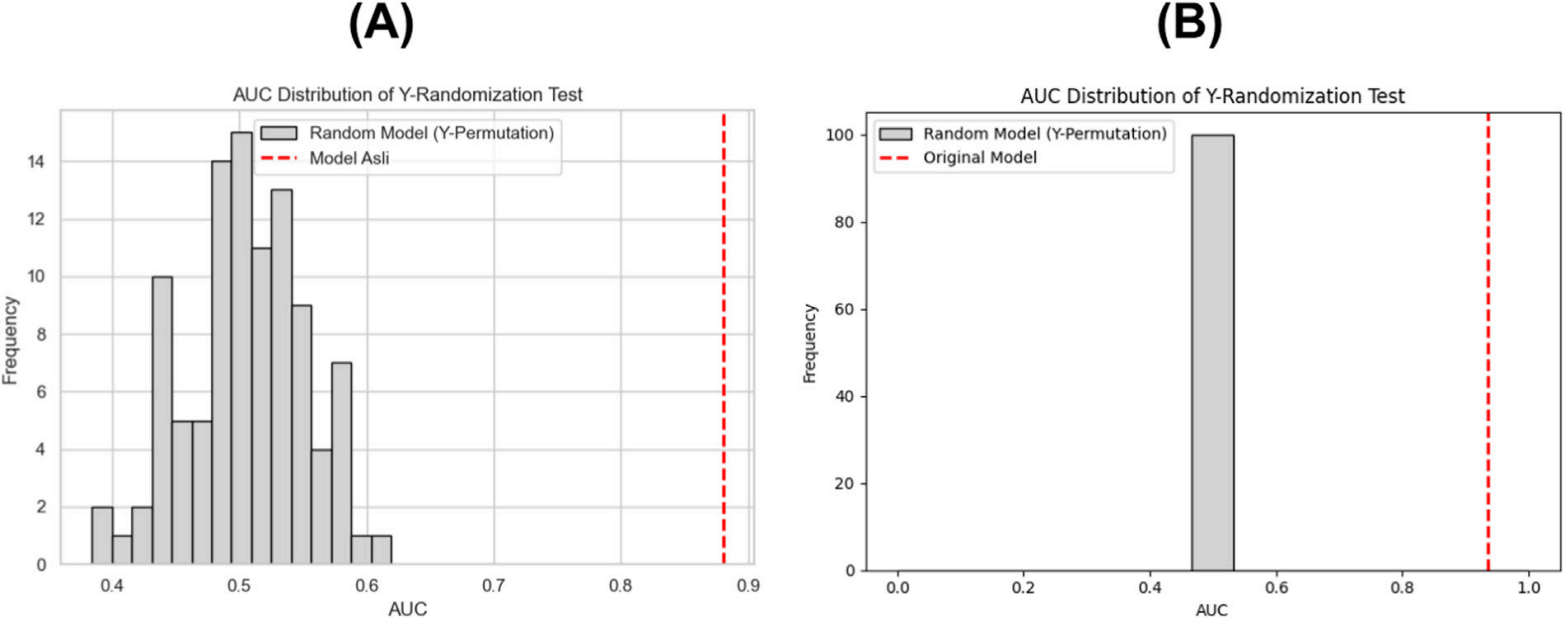
Y-randomization validation of **(A)** the first-layer AdaBoost model using IFP features, and **(B)** the second-layer Random Forest model using chemical descriptor features.

The distribution of AUC values obtained from the permuted and original models is depicted in Figure 3A, where the grey histogram bars represent the randomized models and the red dashed line denotes the original model’s AUC.

In the second-layer model, based on chemical descriptor features and developed using the Random Forest algorithm, the classification performance also remained robust.

The model achieved an AUC of 0.8802, CA of 0.7964, precision of 0.7973, recall of 0.7964, F1-score of 0.7964, and an MCC of 0.5934 (Table 2). A similar Y-randomization test (100 permutations) yielded an average AUC of 0.5039 ± 0.02, whereas the original model maintained an AUC of 0.8802, yielding a permutation-based *p*-value of 0.0099 (p < 0.05).

**TABLE 2:** Performance metrics of the second-layer Random Forest model based on chemical descriptor features.

Metrics	Value
Area under the ROC curve (AUC)	0.8802
Classification accuracy (CA)	0.7964
Precision	0.7973
Recall (sensitivity)	0.7964
F1-score	0.7964
Matthews correlation coefficient (MCC)	0.5934

This finding confirms that the Random Forest model effectively learns true structure activity relationships (SARs) within the chemical descriptor feature space rather than spurious patterns. The comparative performance distribution between the original and randomized models is shown in Figure 3B, where the red dashed line marks the original model’s AUC.

Overall, both classification layers demonstrated statistically significant and reproducible predictive performance, establishing the robustness of the proposed multi-layer ML framework in modeling peptide bioactivity based on IFP and chemical descriptor features. Having confirmed the statistical robustness and validity of both classification layers, further analyses were carried out to explore the interpretability of each model using SHAP-based feature attribution, aiming to uncover the most influential structural and physicochemical factors driving the observed predictive behavior

#### 3.1.2 Model explainability and validation (SHAP analysis)

**FIGURE 4:**
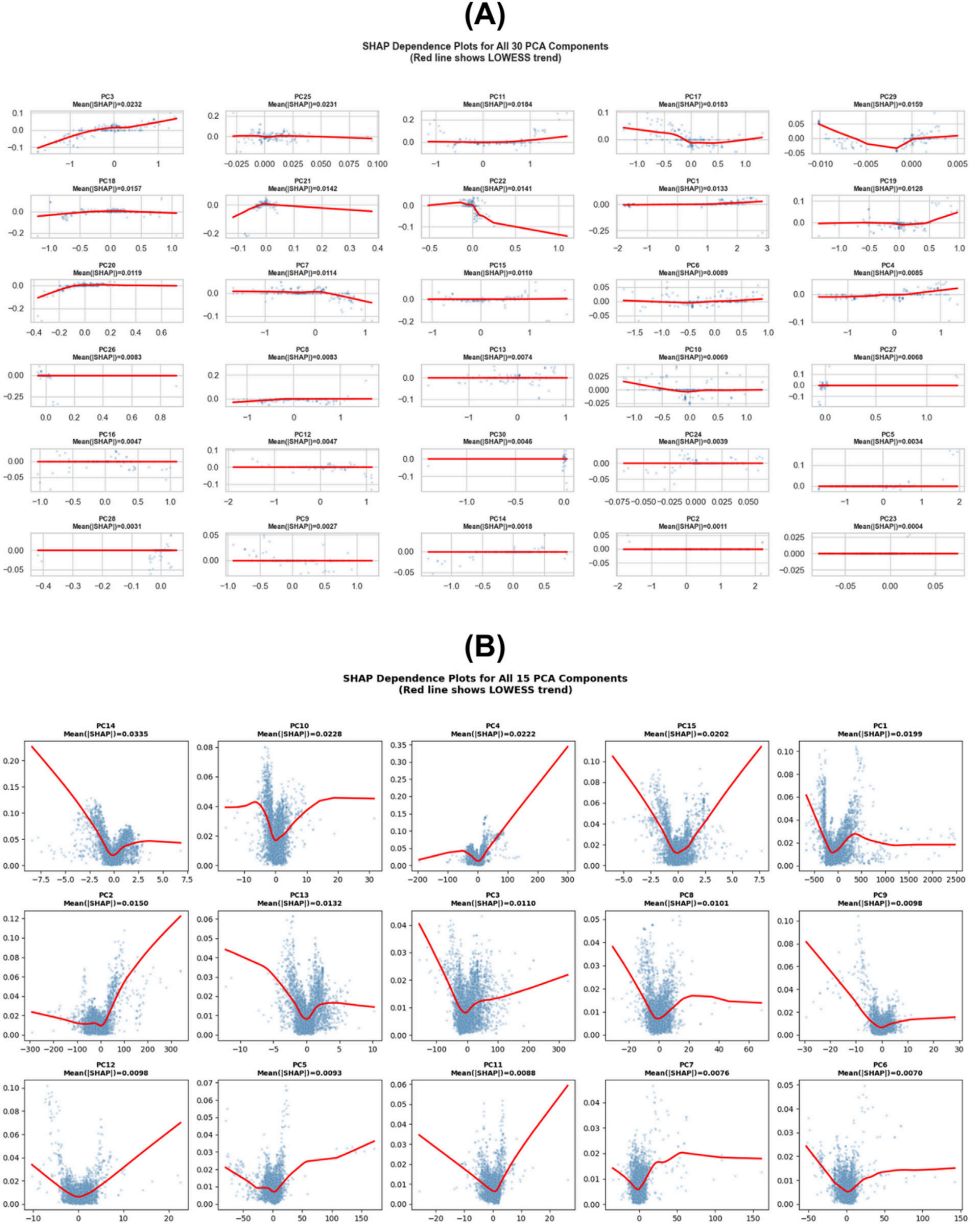
SHAP dependence plots for **(A)** all 30 PCA components used in the AdaBoost model (first-layer), and **(B)** all 15 PCA components used in the Random Forest model (second-layer).

The SHAP dependence analysis provided a comprehensive interpretation of how each principal component (PC) contributed to the AdaBoost classification model (Figure 4A). Among the 30 PCA-derived features, PC3, PC25, and PC11 exhibited the highest average SHAP magnitudes (0.0232, 0.0231, and 0.0184, respectively), suggesting that these latent molecular descriptors exerted the strongest influence on the predicted activity outcomes. The dependence plots demonstrate nonlinear and asymmetric relationships between individual PC values and SHAP impacts, indicating that changes in specific principal components led to either positive or negative shifts in predicted activity probability depending on their structural information.

For example, PC3 showed a monotonic increase in SHAP values with increasing component score, implying a direct relationship between this latent feature and model confidence toward active class prediction. Meanwhile, PC25 and PC11 exhibited more complex, nonlinear effects, suggesting interacting latent features that jointly modulate model output. Intermediate components such as PC17, PC29, and PC1 also showed moderate SHAP contributions (|SHAP| ≈ 0.015–0.013), reinforcing that the predictive signal is distributed across multiple latent variables, not limited to a few dominant axes.

Lower-ranked components (e.g., PC16–PC30) exhibited near-zero SHAP values, indicating minimal influence on classification decisions.

Overall, the SHAP analysis confirms that the AdaBoost model learned chemically meaningful and statistically consistent patterns, while PCA successfully preserved essential structure–activity information rather than introducing noise or redundancy. The SHAP dependence analysis of the Random Forest model provided detailed insights into the relative importance and interaction behavior of PCA-derived chemical descriptor features (Figure 4B).

Among the 15 principal components used as inputs, PC14, PC10, and PC4 exhibited the highest mean absolute SHAP values (0.0335, 0.0228, and 0.0222, respectively), indicating that these latent variables contributed the most to model predictions of IC_50_ activity classes. The dependence plots revealed several distinct nonlinear and asymmetric patterns, showing that changes in certain principal components could either increase or decrease SHAP values depending on their underlying chemical variance.

For instance, PC14 demonstrated a strong positive correlation between component score and SHAP value, implying that molecules with higher PC14 scores were more likely to be predicted as highly active. By contrast, PC10 and PC4 displayed non-monotonic behaviors, where mid-range component values produced maximum SHAP impacts, suggesting localized feature interactions in chemical descriptor space. Lower-ranked components (e.g., PC7–PC15) showed smaller yet interpretable SHAP contributions, reinforcing that the Random Forest model learned from a distributed set of latent molecular features rather than relying on a single dominant variable. This result also supports the effectiveness of PCA in dimensionality reduction retaining chemically informative components while minimizing redundancy.

Overall, the SHAP analysis confirms that the second-layer Random Forest model identified statistically robust and chemically meaningful structure–activity relationships, capturing generalizable descriptors that explain variations in peptide IC_50_ values beyond noise or random correlation. Beyond overall performance, SHAP-based interpretability analysis was conducted to elucidate which PCA-derived features most strongly influenced model predictions in both classification layers (Figure 4). The resulting dependence plots revealed nonlinear and chemically meaningful feature–response relationships, confirming that the models learned interpretable structure–activity patterns rather than noise-driven correlations.

To further interpret the chemical significance of the most influential components identified by SHAP, PCA loading analyses were conducted separately for each classification layer. This step links latent PCs to tangible chemical or structural features, providing mechanistic insight into model behavior.

**FIGURE 5:**
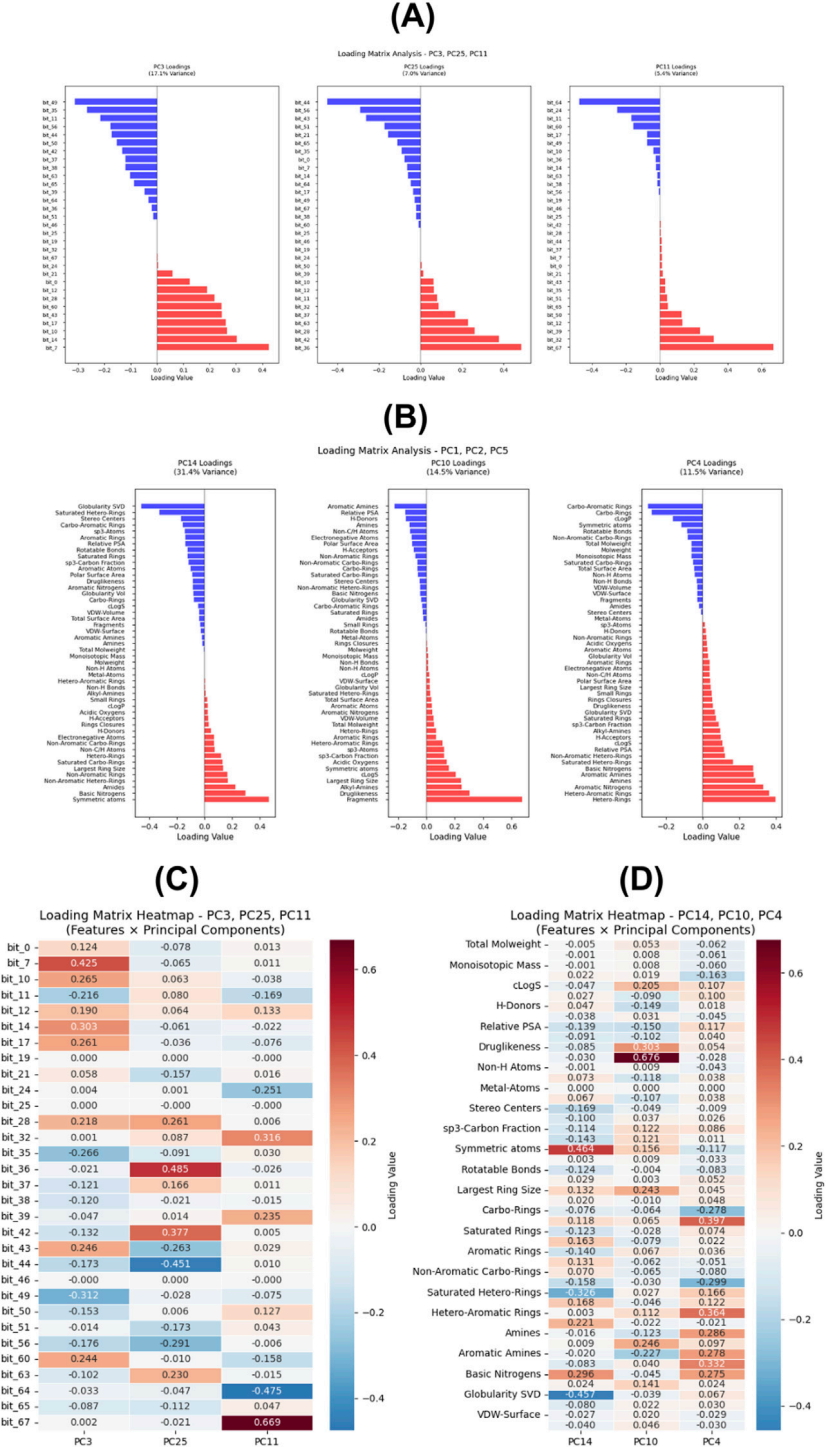
Principal component loading plots and heatmaps of feature contributions in the two-layer ML models. **(A)** PCA loading plots of top components (PC3, PC25, PC11) in the first-layer AdaBoost model based on interaction fingerprints. **(B)** PCA loading plots of top components (PC14, PC10, PC4) in the second-layer Random Forest model based on physicochemical descriptors. **(C and D)** Heatmaps of PCA loading coefficients showing clusters of co-varying features across interaction fingerprints **(C)** and physicochemical descriptors **(D)**.

The loading plots (Figure 5A) revealed that PC3, PC25, and PC11 were dominated by fingerprint bits such as bit_7, bit_36, and bit_67, which correspond to distinct molecular substructures associated with hydrogen bonding, aromatic stacking, and peptide–target interactions. Positive loadings (e.g., bit_7 = +0.42) were related to active peptide patterns, whereas negative loadings (e.g., bit_49 = −0.31) indicated structural motifs linked to inactivity. These findings confirm that AdaBoost captured specific interaction-level information that distinguishes active from inactive peptides.

The global relationship among all fingerprint bits is visualized in the heatmap (Figure 5C), which shows that correlated fingerprints cluster together, suggesting the model recognizes co-occurring substructural motifs rather than isolated features.

For the second-layer Random Forest model, the loading plots (Figure 5B) identified PC14, PC10, and PC4 as key components dominated by global molecular descriptors such as Symmetric Atoms (+0.46), Globularity SVD (−0.45), Hetero-Rings (+0.39), and Druglikeness (+0.30). These descriptors highlight that peptides with higher symmetry, moderate hetero-ring presence, and favorable topological complexity tend to show greater bioactivity probabilities. In contrast, more globular and irregularly structured molecules contributed negatively to model confidence.

The accompanying heatmap (Figure 5D) visualizes the overall distribution of descriptor loadings across components. Clear feature clusters are visible for ring-related and aromaticity-based descriptors, indicating that Random Forest learned chemically coherent feature groupings that reflect underlying structure–activity relationships.

#### 3.1.3 Applicability domain (AD) and model generalization

**FIGURE 6:**
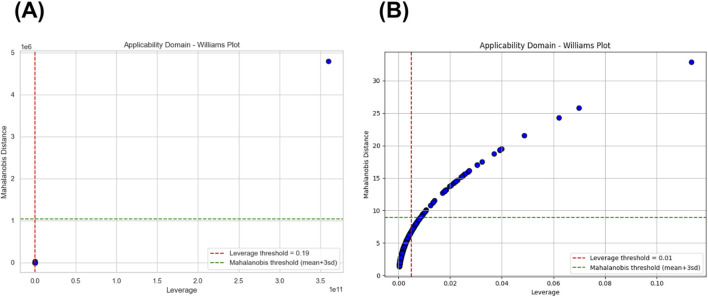
Williams plot of: **(A)** The first-layer AdaBoost model based on PCA-transformed IFP features. The plot shows leverage (x-axis) *versus* Mahalanobis distance (y-axis). The red dashed line indicates the leverage threshold (h* = 0.19), and the green dashed line marks the Mahalanobis distance limit (*mean + 3SD*). Most compounds are within both boundaries, with only one structural outlier detected. **(B)** The second-layer Random Forest model based on PCA-transformed chemical descriptor features. The red dashed line represents the leverage threshold (h* = 0.01), and the green dashed line denotes the Mahalanobis distance threshold (*mean + 3SD*). All compounds fall within the AD region, confirming that predictions are made within a chemically reliable and generalizable domain.

The applicability domain (AD) analysis was conducted to evaluate the structural reliability and generalization capability of both classification layers. The AD was examined using Williams plots, which integrate leverage (h*) and Mahalanobis distance (MD) criteria to identify potential outliers or structurally influential compounds. The quantitative summary of AD metrics for each model is presented in Table 3, while the graphical representations are illustrated in Figure 6.

**TABLE 3:** Application Domain (AD) metrics for both classification models.

Model layer	Algorithm	Feature type	Leverage threshold (h*)	Mahalanobis distance threshold (mean + 3SD)	Outliers detected	Interpretation
First layer	AdaBoost	PCA-transformed interaction fingerprint (IFP) features	0.19	Mean + 3SD	1 compound	Model operates within a well-defined applicability domain; one compound identified as structurally distinct from the training domain.
Second layer	Random forest	PCA-transformed chemical descriptor features	0.01	Mean + 3SD	None	All compounds fall within the AD boundaries; predictions are chemically consistent and generalizable.

In the first-layer AdaBoost model, which utilized PCA-transformed IFP features, the leverage threshold (h*) was 0.19. As shown in Figure 6A, the vast majority of compounds were located within the defined AD boundaries, indicating that predictions were made on structurally representative samples. Only one compound exceeded both the leverage and Mahalanobis distance thresholds, suggesting that it represents a structurally unique tripeptide distinct from the main chemical domain. Overall, the model operated within a well-defined and statistically robust applicability domain, ensuring reliable predictions for most compounds.

For the second-layer Random Forest model, based on PCA-transformed chemical descriptor features, the leverage threshold (h*) was 0.01, and no compounds exceeded the predefined boundaries (Figure 6B). All predictions were made within the valid chemical domain, demonstrating that the model generalizes well to unseen data and avoids extrapolation beyond the trained chemical space. Collectively, these results confirm that both classification layers exhibit structural consistency, robustness, and generalizability, reinforcing confidence in their predictive outputs.

Overall, the Y-randomization, SHAP interpretability, and AD analyses collectively validate that the proposed multilayer AdaBoost–Random Forest framework achieves high predictive accuracy while maintaining interpretability and domain reliability across the chemical space.

### 3.2 QSAR model evaluation and external data prediction

The regression-based QSAR models showed strong predictive performance for both bioactivity and lipophilic efficiency. The IC_50_ prediction model achieved an *R* ^2^ of 0.955 and RMSE of 0.231, with a regression equation (y = 0.9329*x* + 0.0007) based 1,655 training ligands, closely matching the ideal line, indicating reliable prediction of inhibitory potency across diverse tripeptides (Supplementary Figure S2A). Similarly, the LELP values calculated from IC_50_ prediction yielded an excellent model performance, with an *R* ^2^ of 0.994 and RMSE of 0.469, showing a strong linear fit (y = 0.9742*x* + 0.22) based on 2,178 training ligands, suggesting accurate estimation of lipophilicity-related efficiency (Supplementary Figure S2B). Together, these models support the early identification of peptide candidates with both high potency and favorable pharmacokinetic profiles. Within 628 peptides, 88 peptides showing ideal features of LELP (−5<LELP<5). These 88 peptides were subsequently subjected to molecular docking and re-scoring MM/GBSA to extract the best hits.

### 3.3 Druglikeness assesment

**FIGURE 7:**
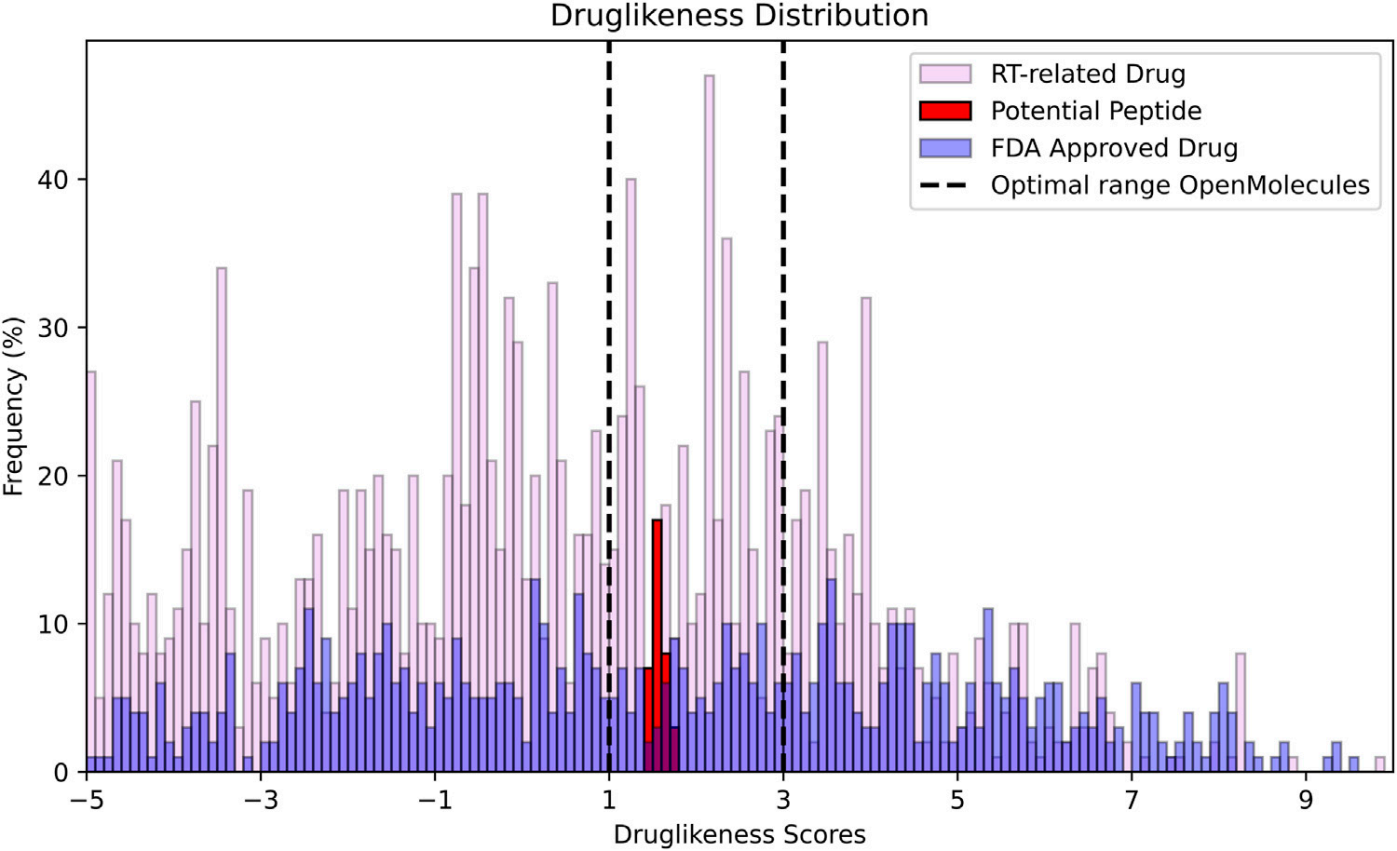
The distribution of druglikeness scores was evaluated across three categories, potential peptides (35 molecules), reverse transcriptase-related drugs (2,449 molecules), and FDA-small molecules approved drugs (865 molecules). The frequency of compounds is expressed as a percentage on the y-axis, while the x-axis represents the druglikeness scores. The black dotted line indicates the optimal range of druglikeness (UNAIDS, 2024; Margolis et al., 2014; Shi et al., 2016) based on the frequency of the majority of druglikeness values being positive in OpenMolecules.

The distribution of druglikeness scores shows (Figure 7) that the 35 potential peptides are narrowly clustered within the optimal range of 1–3. This indicates that the peptides possess a favorable druglike profile with minimal variability. In contrast, RT-related drugs (pink) and FDA-approved small molecules drugs (blue) display broader distributions, with many compounds extending beyond the optimal druglikeness window. Although FDA-approved small molecules drugs include several compounds within the 1–3 range, their overall distribution is more dispersed. RT-related drugs show the widest spread, including a large fraction with negative druglikeness values. These findings suggest that, relative to RT-related and FDA-approved drugs, the screened peptides are more consistently positioned within the optimal druglikeness region, highlighting their potential as promising drug candidates.

### 3.4 *In silico* verification

#### 3.4.1 Molecular docking assessment

The screening of peptides using machine learning and QSAR model was then further verified using molecular docking assessment. As shown in Table 4, molecular docking using AutodockVina, Autodock4, and MM/GBSA re-scoring showed that the five peptides (FHW, HFW, HHW, WHH, HYF) have lower binding energy than Nevirapine. The lowest binding energy was shown by the FHW peptide with the binding energy of −63.50 kcal/mol using MM/GBSA, −11.47 kcal/mol using Autodock4, and -9.20 kcal/mol using AutodockVina. Meanwhile, the highest binding energy was shown by Nevirapine with the binding energy of −44.97 kcal/mol using MM/GBSA and −9.08 kcal/mol using Autodock4. However, in Autodock Vina, Nevirapine showed the lowest binding energy (−10.69 kcal/mol). These consistent results across docking protocols and MM/GBSA re-scoring indicate that the FHW peptide possesses the most favorable binding affinity among all tested candidates.

**TABLE 4:** Comparison of binding energy from the potential peptides with the Nevirapine.

Ligands	Energy (kcal/mol)		
	AutoDock vina	AutoDock4	MM-(GB)sa
Nevirapine	−10.69	−9.08	−44.97
FHW peptide	−9.20	−11.47	−63.50
HFW peptide	−9.20	−10.52	−63.26
HHW peptide	−8.60	−10.32	−62.69
WHH peptide	−8.80	−10.71	−59.76
HYF peptide	−8.90	−9.48	−59.41

**FIGURE 8:**
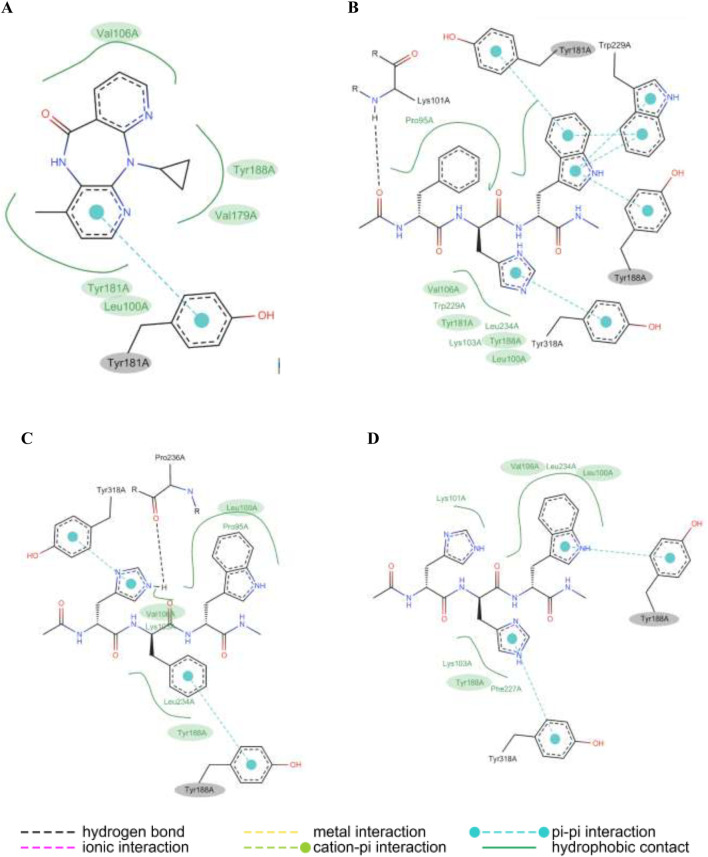
The 2D visualization of reverse transcriptase with **(A)** nevirapine **(B)** FHW peptide **(C)** HFW peptide **(D)** HHW peptide.

The 2D visualization of molecular docking showed interaction of RT-HIV with Nevirapine, HHW peptide, HFW peptide, and FHW peptide. As shown in Figure 8A, Nevirapine interacts with RT-HIV in the five residues, including Leu100, Val106, Val179, and Tyr188 by hydrophobic contacts, also Tyr188 with both hydrophobic contacts and hydrogen bonds. HFW and HHW peptides showed three same residues with Nevirapine, including Leu100, Val106, and Tyr188 by hydrophobic contacts (Figures 8C,D, respectively). Meanwhile, FHW peptide in Figure 8B shows four same residues with Nevirapine, including Leu100, Val106, Tyr181, and Tyr188 by hydrophobic contacts. Tyr181 and Tyr188 of the FHW peptide also interact by hydrogen bond with RT-HIV. The 3D visualization demonstrates that all potential tripeptides can bind within the same region as the control compound Nevirapine (see Supplementary Figure S4).

#### 3.4.2 Pocket assessment similarity

To evaluate the binding pocket similarities between candidate peptides and Nevirapine (NVP), several cavity parameters were analyzed, including the number of hydrogen bond acceptors and donors, pocket depth, hydrophobicity, and other physicochemical characteristics. The pocket associated with NVP exhibited 11 hydrogen bond acceptors and eight donors, a depth of 15.47 Å, and a hydrophobicity index of 0.79 (Table 5). In comparison, all candidate peptides (FHW, HFW, and HHW) showed higher values for both acceptors (Sander et al., 2015; Tien et al., 2013; Istyastono et al., 2020; Trott and Olson, 2010; Radifar et al., 2012) and donors (Baassi et al., 2023; Mysinger et al., 2012). The peptide HHW had the highest number of acceptors (Radifar et al., 2012). Pocket depth was also deeper for all peptides (18.07–20.77 Å) than for NVP, with HFW showing the deepest cavity at 20.77 Å. Hydrophobicity values were slightly lower for the peptides (0.73–0.75) compared to NVP (0.79). Metal interactions were absent across all pockets. Regarding the number of protein heavy atoms and the pocket surface and volume, peptides generally displayed higher surface area (558.49–589.81 Å^2^) and pocket volume (763.03–856.92 Å^3^) compared to NVP (487.66 Å^2^ and 640.74 Å^3^, respectively.

**TABLE 5:** Pocket parameter analysis.

Pocket parameter	Nevirapine	FHW peptide	HFW peptide	HHW peptide
Acceptors	11.00	14.00	17.00	18.00
Depth (Å)	15.47	18.07	20.77	19.68
Donors	8.00	12,00	12.00	11.00
Hydrophobicity	0.79	0.75	0.75	0.73
Metal	0.00	0.00	0.00	0.00
Protein heavy atoms	156.00	189.00	211.00	201.00
Surface (Å^2^)	506.77	621.62	792.15	731.58
Surface-volume ratio	1.36	1.10	1.02	1.12
Volume (Å^3^)	372.22	564.22	778.24	650.75

#### 3.4.3 Quantum mechanical analysis

The HOMO-LUMO energy gap (ΔE) and frontier molecular orbital (FMO) play critical roles in understanding molecular reactivity, chemical softness, and hardness, particularly within the framework of DFT applications in drug design and biochemistry. The HOMO and LUMO are essential descriptors for predicting chemical behavior, reactivity, and biological activity of compounds. Frontier molecular orbital theory, initially proposed by Kenichi Fukui, asserts that the interaction between the HOMO and LUMO dictates the potential reactivity of a molecule. Reactivity is typically governed by electron transfer, where the HOMO acts as an electron donor and the LUMO serves as an electron acceptor. This paradigm enhances our understanding of various chemical processes and can be quantitatively analyzed using DFT methods, which provide insights into molecular structure, stability, and global reactivity descriptors such as chemical hardness and softness (Chidiebere et al., 2021; Li et al., 2021).

The HOMO–LUMO energy gap (ΔE) is an important quantum chemical parameter that describes the chemical reactivity and stability of a molecule. A smaller energy gap value indicates that the molecule requires less energy to excite an electron from the HOMO to the LUMO, suggesting higher chemical reactivity and molecular softness. This relationship is well established in FMO theory, where compounds with narrow ΔE values are considered more reactive and exhibit a greater tendency to participate in charge transfer or electron delocalization processes (Parr and Pearson, 2002; Chattaraj et al., 2006; Pearson, 2005). Therefore, the low ΔE values observed in the modified compounds imply enhanced reactivity and potential biological interaction due to increased electron mobility within the molecular orbitals. Moreover, the concepts of chemical softness and hardness, defined by Pearson’s HSAB (Hard and Soft Acids and Bases) theory, correlate directly with the HOMO and LUMO energies. Hard acids and bases have high ionization potentials and low electron affinities, while soft acids and bases have lower ionization potentials and higher electron affinities. These principles allow chemists to predict the outcome of reactions involving different reagents based on their relative softness or hardness (Sun et al., 2015; Kohagen et al., 2019; Ayun et al., 2022). Thus, computational chemistry employing DFT and frontier molecular orbital theory provides a robust framework for predicting the behavior of potential drugs and their interactions.

**FIGURE 9:**
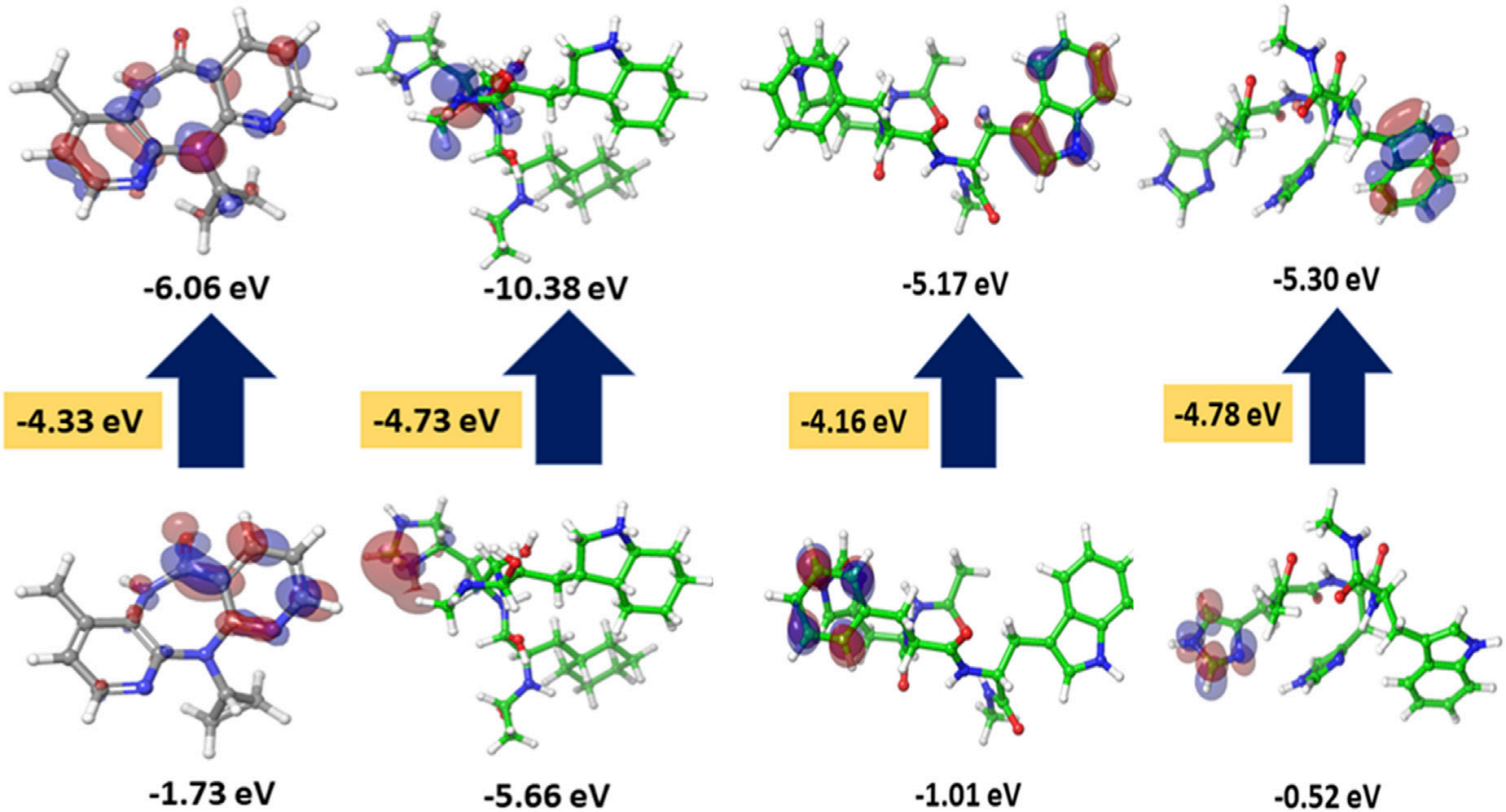
DFT analysis of HOMO-LUMO orbitals using b3LYP-D3 for Nevirapine, FHW peptide, HFW peptide, and HHW peptide.

To evaluate the stability and reactivity of the studied compounds, the energy difference between the HOMO and LUMO orbitals was calculated using DFT analysis. This computational method provides valuable insight into the molecule’s electronic structure and facilitates the evaluation of its potential pharmacological activity. Table 6 presents the calculated HOMO and LUMO energies, along with other global reactivity descriptors such as ionization potential (I), electron affinity (A), chemical potential (μ), electronegativity (χ), hardness (η), softness (S), and electrophilicity index (ω). The HOMO energy reflects the electron-donating capability, whereas the LUMO energy signifies the electron-accepting ability. The key FMO energies of the four ligands were computed using DFT at the B3LYP level and refined with B3LYP-D3. The 3D orbital plots are shown in Figure 9. The calculations revealed that the HOMO and LUMO orbitals were mainly localized in the benzene ring region. Among the compounds, the FHW peptide exhibited the lowest HOMO value (−10.38 eV), indicating higher stability upon electron loss compared to other peptides and Nevirapine. Similarly, the FHW peptide also showed the lowest LUMO value, suggesting a stronger tendency to accept electrons. The resulting HOMO–LUMO gap (ΔE) of the FHW peptide was 4.73 eV, while the HHW peptide had a ΔE of 4.78 eV. These relatively low energy-gap values indicate that both peptides are softer and more chemically reactive, allowing electrons to move more freely and facilitating better orbital interactions with target residues during binding.

**TABLE 6:** Calculation results of frontier molecular orbital (FMO).

Ligands	Homo (eV)	Lumo (eV)	Energy gap (eV)	I (eV)	A (eV)	μ (eV)	χ (eV)	η (eV)	S (eV)	ω (eV)
NVP (ref drug)	−6.06	−1.73	−4.33	6.06	1.73	−3.90	3.90	2.16	0.23	3.51
Peptide_FHW	−10.38	−5.66	−4.73	10.38	5.66	−8.02	8.02	2.36	0.21	13.60
Peptide_HFW	−5.17	−1.01	−4.16	5.17	1.01	−3.09	3.09	2.08	0.24	2.30
Peptide_HHW	−5.30	−0.52	−4.78	5.30	0.52	−2.91	2.91	2.39	0.21	1.77

Abbreviations: I, Ionization potential; A, Electron affinity; μ, Chemical potentials; χ, Electronegativity; η, Hardness; S, Softness; ω, Electrophilicity index.

Based on these findings, the FHW peptide demonstrates the highest electrophilicity index among the four ligands, indicating superior electrophilic character relative to Nevirapine. This enhanced reactivity, supported by the low ΔE values, aligns with the theoretical framework established by FMO and HSAB theories, confirming that compounds with smaller HOMO–LUMO gaps are indeed softer and more reactive (Parr and Pearson, 2002; Chattaraj et al., 2006; Pearson, 2005). Therefore, the observed data validate that the FHW peptide possesses favorable electronic properties, potentially contributing to stronger binding affinity with the target protein.

#### 3.4.4 Permeability prediction

**FIGURE 10:**
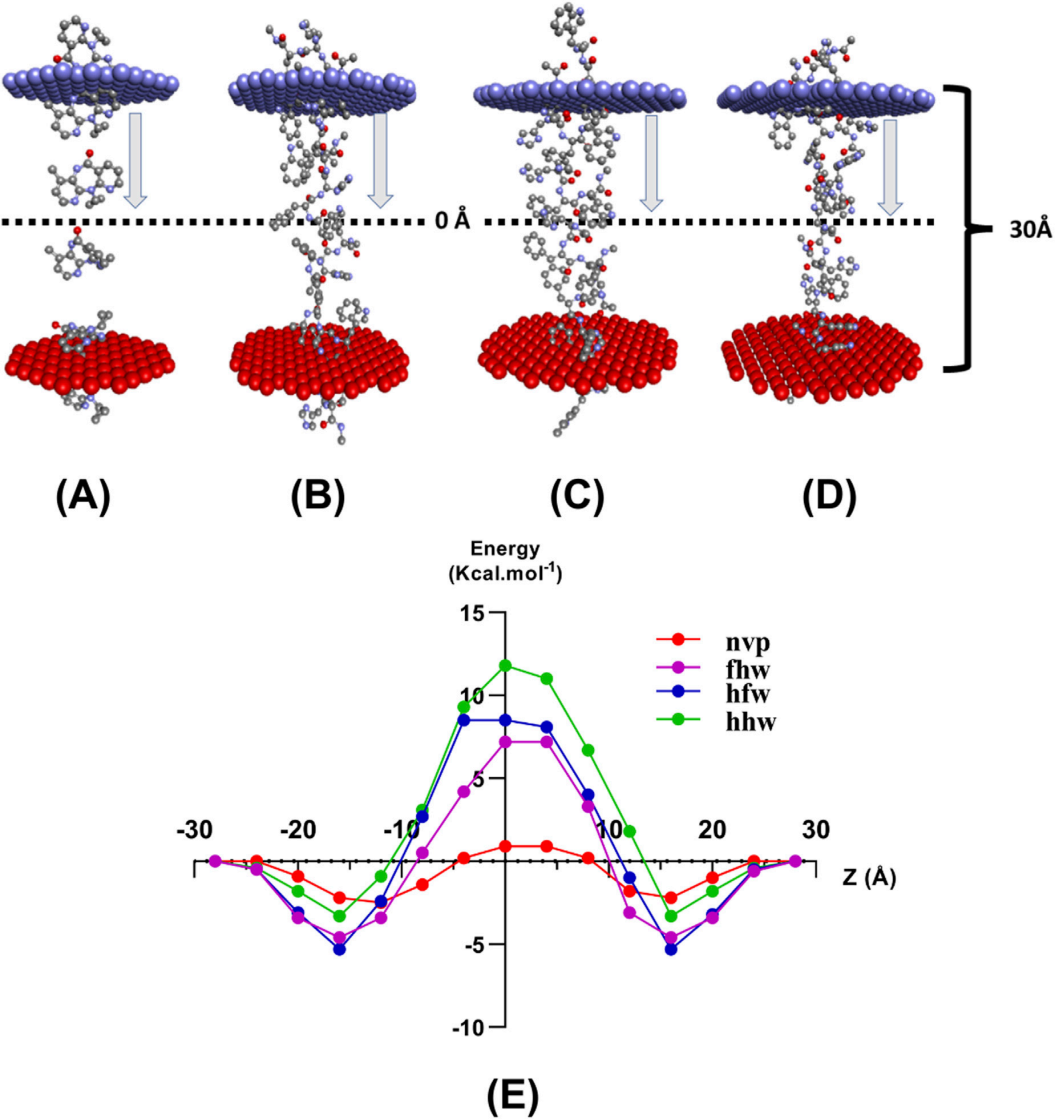
Estimated BLM permeability pathways across the black lipid membrane (BLM) in **(A)** Nevirapine, **(B)** FHW peptide, **(C)** HFW peptide, and **(D)** HHW peptide with **(E)** plot of the energy gap during ligand penetration of the BLM membrane.

The permeability profiles, as predicted by PerMM, indicated that FHW and HFW peptides exhibited strong passive transport across the artificial lipid bilayer. Energy distribution analyses (Figure 10) revealed consistent negative energy profiles for FHW peptide during membrane translocation, signifying favorable interactions with the lipid environment. Conformational flexibility during bilayer crossing further enhanced FHW’s efficiency, as shown in Figure 10A. Figure 10E shows the energy profiles of FHW peptide and other peptides during membrane permeability studies.

#### 3.4.5 All atomics dynamic simulation

**FIGURE 11:**
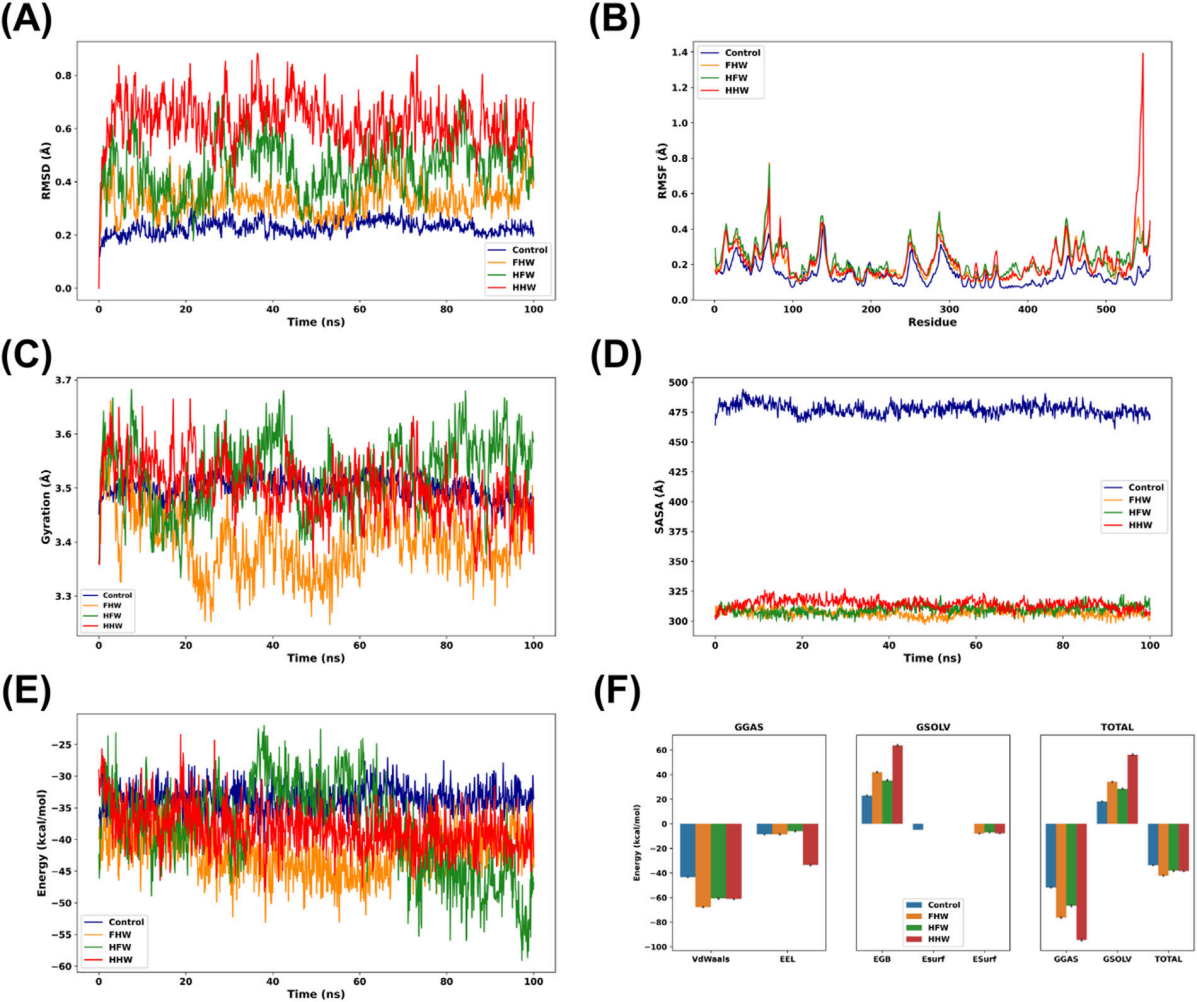
MD analysis using **(A)** RMSD, **(B)** RMSF, **(C)** Radius of Gyration, **(D)** Solvent-Accessible Surface Area, **(E)** MMGBSA, **(F)** MMGBSA Energy Components.

The MD trajectory analysis revealed that the FHW tripeptide exhibited increased structural stability in complex with HIV-1 reverse transcriptase compared to Nevirapine (NVP) as control, as shown by the protein’s backbone Root Mean Square Deviation (RMSD) (Figure 11A). This suggests that FHW (orange) forms a more compatible and stable binding within the active pocket to achieve a stable conformation after an initial adjustment, fluctuating similarly to the NVP control (dark blue). In contrast, HFW (green) and HHW (red) exhibited significantly higher fluctuations, indicating greater instability and movement within the active site. Additionally, the Root Mean Square Fluctuation (RMSF) analysis (Figure 11B) showed a notable decrease in atomic fluctuations for FHW, particularly during the early and middle phases of the simulation, when compared to both HFW and HHW. This reduced flexibility implies a more stable and tight interaction between FHW and the target protein throughout the simulation period.

Furthermore, the radius of gyration (Rg) (Figure 11C), which reflects the protein’s folding behavior, indicated that the FHW complex maintained more consistent folding with minimal fluctuations relative to the NVP-bound complex. This observation supports the hypothesis that FHW binding does not induce significant destabilization of the protein structure. HHW exhibits high fluctuations in RMSD, RMSF, and radius of gyration values, indicating that the formed complex still has the flexibility enabling conformation changing of the target protein structure. This high fluctuation also indicates that the protein’s functionality is still maintained because it still allows the protein structure to change shape to adjust to the required substrate, which indicates that the stiffness that occurs due to HHW attachment is still low compared to the peptide and control.

Analysis of the complex’s compactness using the Solvent-Accessible Surface Area (SASA) (Figure 11D) showed that the FHW-bound complex consistently maintained a lower SASA value compared to the control and other peptides. This suggests that FHW binding induces a more compact protein conformation, likely by shielding a larger portion of the protein surface from the solvent and further indicating a stable binding event.

Finally, to quantify the binding affinity, the binding free energy was calculated using the MM/GBSA method (Figure 11E). The FHW complex exhibited the lowest (most negative) average binding free energy (ΔG), indicating a significantly stronger binding affinity to the HIV-1 reverse transcriptase compared to the NVP control, as well as the HFW and HHW peptides. The energy component analysis (Figure 11F) reinforces this finding, showing that the strong binding of FHW was primarily driven by substantial negative contributions from both van der Waals (ΔG_vdW_) and electrostatic (ΔG_ele_) interactions. These favorable interactions effectively overcome the unfavorable solvation energy (ΔG_solv_), resulting in the most favorable total binding energy among the tested compounds.

#### 3.4.6 PCA and DCCM analysis

**FIGURE 12:**
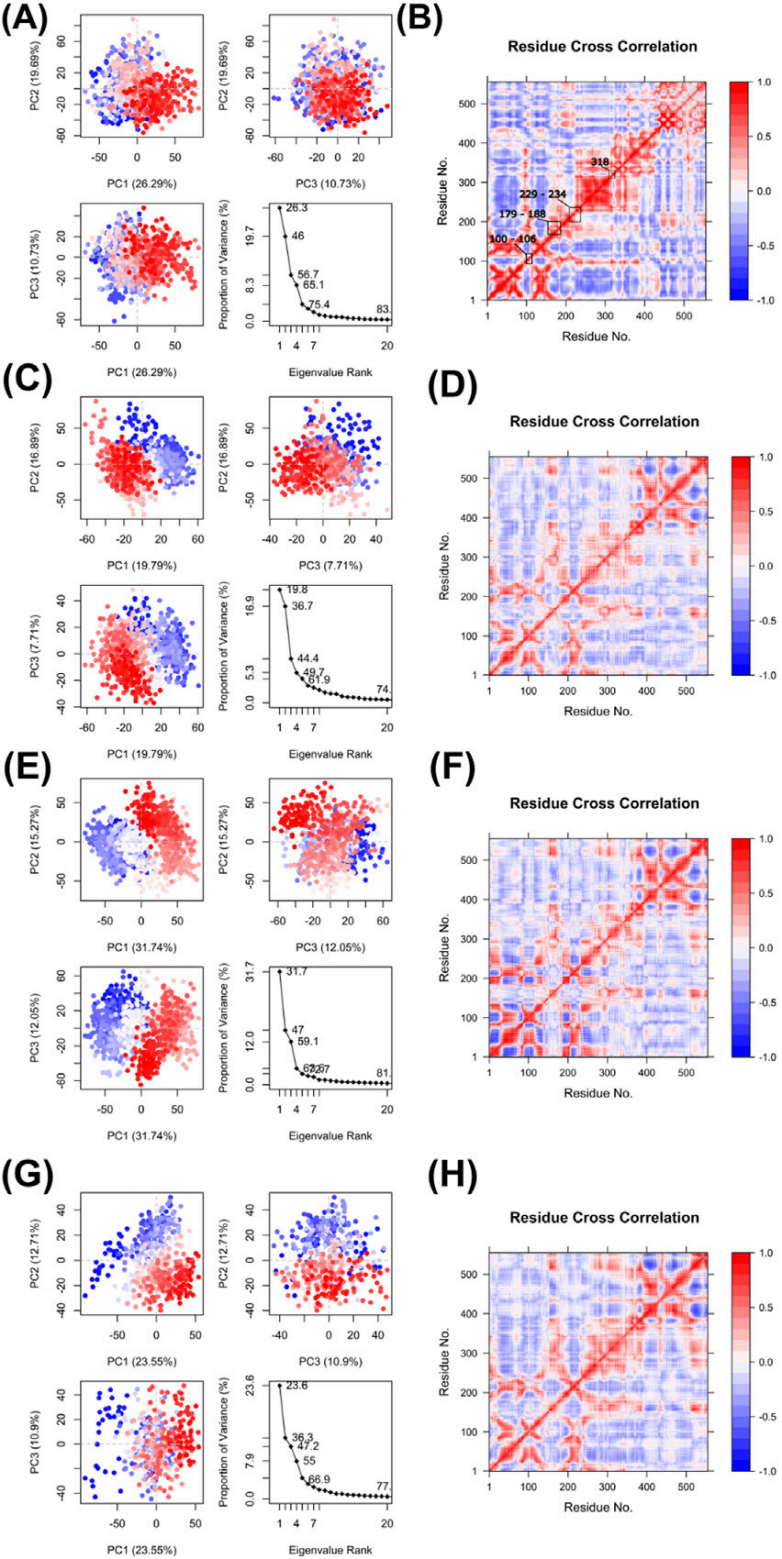
Post-analysis of MD results which include **(A)** PCA of Nevirapine **(B)** DCCM of Nevirapine **(C)** PCA of FHW peptide **(D)** DCCM of FHW peptide **(E)** PCA of HFW peptide **(F)** DCCM of HFW peptide **(G)** PCA of HHW peptide **(H)** DCCM of HHW peptide.

The dynamics of RT in complex with Nevirapine and the three modelled peptides were evaluated using PCA, as shown in Figure 12. Each complex demonstrated distinct motion patterns in the principal component space, reflecting their dynamic stability and conformational variance. The eigenvalue plots further confirmed that most of the motion was captured within the first few principal components for all complexes. Nevirapine complex (Figure 12A) displayed a moderate spread in PC1 and PC2, with the first two components accounting for 28.3% and 19.7% of the total motion, respectively, suggesting controlled but noticeable conformational fluctuation. FHW peptide complex (Figure 12C) showed a tighter clustering pattern, indicating reduced flexibility. PC1 and PC2 accounted for 19.79% and 16.89% of the motion, reflecting a more rigid and stable conformation upon peptide binding. FHW peptide complex (Figure 12E) had broader distributions, especially in PC1 and PC2 accounted for 31.74% and 12.05%, indicating greater conformational sampling and flexibility, potentially reflecting more dynamic interactions within the binding site. The eigenvalue plots further confirmed that most of the motion was captured within the first few principal components for all complexes. HHW peptide complex (Figure 12G) had low distributions compare to Nevirapine and HFW but still higher than FHW, which have PC1 and PC2 accounted for 23.55% and 12.71%.

The DCCM analyses (Figures 12B,D,F,H) revealed correlated (red) and anti-correlated (blue) motions between residue pairs, both directly (neighboring residues) and indirectly (distant residues), throughout the Reverse Transcriptase protein. In the Nevirapine-bound complex (Figure 12B), strong correlated motions were observed in the central core of the protein, reflecting stable domain communication. The FHW complex (Figure 12D) showed less intense correlated and also anti-correlated patterns, suggesting that peptide binding may reduce long-range residue coupling, leading to a more localized and rigid dynamic profile. In contrast, the HFW and HHW complexes (Figures 12F,H) exhibited widespread correlation changes across several domains, with a higher prevalence of anti-correlated patterns compared to Nevirapine and FHW. This suggests that peptide binding may alter the intrinsic flexibility and coordination of residue motions. Together, these PCA and DCCM analyses provide insight into the impact of peptide binding on the structural dynamics of Reverse Transcriptase, with all potential tripeptides exhibiting stabilized behavior. Among them, FHW displayed greater stability compared to the other peptides as well as the control Nevirapine.

#### 3.4.7 Free energy landscape (FEL)

**FIGURE 13:**
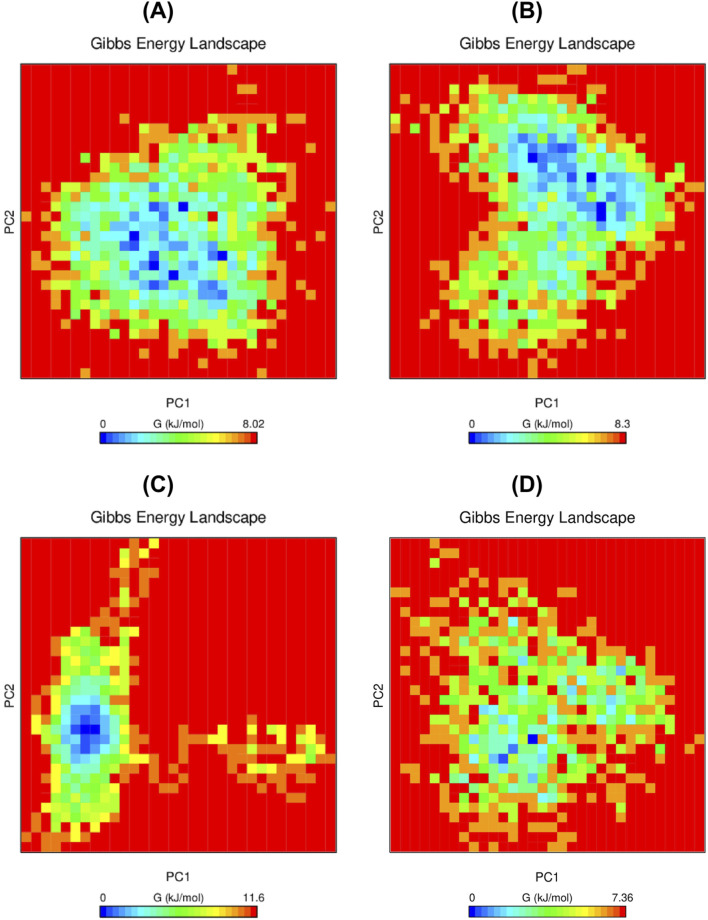
Free Energy Landscape During Simulation **(A)** Nevirapine **(B)** FHW **(C)** HFW **(D)** HHW peptide.

The energy landscape is an approach used to predict the possible conformations of a protein structure, whereby the energy landscape can describe the conformational states in a simulation system and the position of the protein with its minimum (Gibbs) energy. In this study, PC1 and PC2 were used in principal component analysis to describe changes in the system during simulation, with a color gradient from red (unstable conformation) to blue (stable conformation) (Figures 13A–D) (Abdelsattar et al., 2021).

In the FHW RT-peptide complex (Figure 13B), the FEL map (based on PC1–PC2) shows two minimum regions that are wider and steeper on one side compared to the other complex. In addition, the range of FHW minimum energy is close to the NVP range. RT-HFW complex (Figure 13C) displays a single dominant minimum with a relatively concentrated distribution. This describes high thermodynamic stability with low transition barriers, so that the system easily fluctuates around a single main valley without needing to climb a significant barrier. The RT-peptide HHW complex (Figure 13D) is the most shallowly multi-state, with many sub-states of similar depth and low barriers, reflecting high flexibility but without a single steep transition path.

The diagonal elongated pattern with separate blue clusters on RT-FHW indicates the presence of a directed transition pathway that must pass through an energy barrier before the system can transition between states. Physically, this is consistent with a scenario in which ligand binding forces the RT-HIV enzyme into alternative conformational sub-spaces that are not smoothly connected, so that transition between basins requires extra energy (Durojaye et al., 2023). In other words, the RT-peptide FHW complex shows greater conformational changes in the viral protein, indicating a meaningful restructuring of conformation. Consequently, the bound state may become more locked (kinetically stabilized) in one of the basins, even though its overall energy profile is higher than that of complexes with a smoother landscape (Abdelsattar et al., 2021). This finding is consistent with (Durojaye et al., 2023) where antiviral compounds tend to massively alter the conformation of viral proteins.

#### 3.4.8 Steered dynamic simulation

**FIGURE 14:**
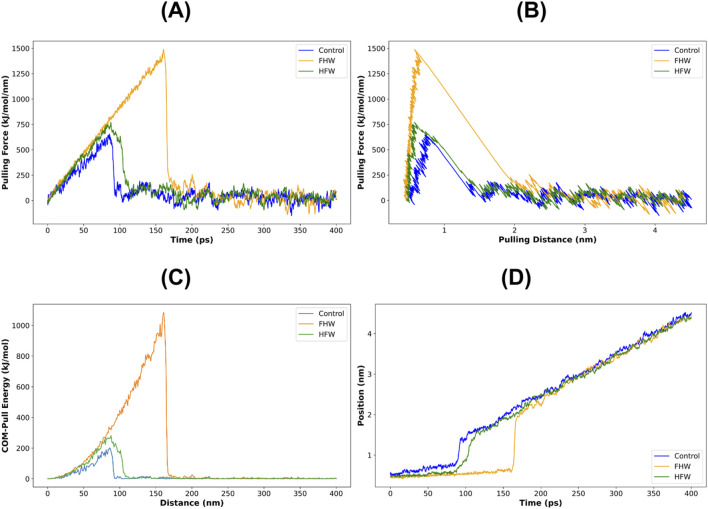
Steered Dynamic Simulation **(A)** pulling force vs. time **(B)** pulling force vs. distance **(C)** COM-Pull vs. distance **(D)** position vs. time (ps) during 400 ps.

The stability and uncoupling mechanisms of the complex formed between FHW and HFW with Reverse Transcriptase, compared to the Nevirapine control, were tested by external stimuli in a steered dynamic simulation. Mechanical stress was applied to check the stability of the interaction of each complex. The tensile force was gradually increased over time, with the FHW peptide requiring the highest tensile force, approximately 1,500 kJ mol^−1^·nm^−1^, and the longest duration, around 150–180 ps, to dissociate from its complex until reaching a separation of approximately 2 Å (Figures 14A,D). This information is also in line with Figures 14B,C, where, due to the large pulling force and energy required to separate FHW from the complex, the resulting distance of the bounce after the complex’s uncoupling was greater than that of Nevirapine and HFW. The control complex had the lowest stability compared to HFW and FHW in this steered dynamic simulation experiment because the pulling force required to separate the complexes was only around 200 kJ/mol, compared to ∼280 and ∼1,100 kJ/mol in HFW and control, respectively.

### 3.5 Pharmacokinetic prediction

Pharmacokinetic properties of the selected peptides (Table 7) were predicted using the pkCSM approach. The peptide derivatives FHW and HFW demonstrate moderate water solubility (−2.903 and −2.904 log mol/L) and limited Caco-2 permeability, with intestinal absorption values of 61.97% and 62.18%, respectively. In contrast, the reference compound NVP displayed lower solubility (−3.968 log mol/L) but markedly higher permeability (1.271 log Papp) and excellent intestinal absorption (97.08%). Both peptides were predicted as P-glycoprotein (P-gp) substrates and inhibitors, indicating potential efflux liability, while NVP was not associated with P-gp interaction. In terms of distribution, the peptides showed extensive tissue distribution (VDss: 0.642 and 0.478 log L/kg) and a very low fraction unbound (∼0.04), suggesting high plasma protein binding and limited free drug availability. Their low blood–brain barrier (log BB < −1.7) and CNS permeability (log PS < −3.5) means limited blood-brain barrier permeability. Conversely, NVP showed lower VDss (0.241 log L/kg) but a higher fraction unbound (0.383), with measurable BBB penetration (log BB = 0.22) and better CNS permeability (−2.848 log PS). Regarding metabolism, FHW and HFW were predicted as CYP3A4 substrate with no any CYP isozyme inhibitor, whereas NVP was not a CYP3A4 substrate and CYP1A2 inhibitor. For excretion parameters, the peptides revealed a higher total clearance (1.079 and 0.911 log mL/min/kg) compared to NVP (0.013 log mL/min/kg) in addition to HFW that was identified as a renal OCT2 substrate.

**TABLE 7:** Prediction of pharmacokinetic properties.

Compound	Absorption					
	Water solubility (log mol.L)	Caco2 permeability (log Papp in 10–6 cm/s)	Intestinal absorption (%)	P-GP substrate	P-GP1 inhibitor	P-GP2 inhibitor
FHW	−2.903	−0.585	61.967	Yes	Yes	Yes
HFW	−2.904	−0.577	62.184	Yes	Yes	Yes
NVP	−3.968	1.271	97.086	No	No	No.

	Distribution			
Compound	VDss (log L/kg)	Fraction unbound (fu)	BBB permeability (log BB)	CNS permeability (log PS)
FHW	0.642	0.04	−1.714	−3.609
HFW	0.478	0.044	−1.729	−3.586
NVP	0.241	0.383	0.22	−2.848

	Metabolism						
	CYP2D6 substrate	CYP3A4 substrate	CYP1A2 inhibitor	CYP2C19 inhibitor	CYP2C9 inhibitor	CYP2D6 inhibitor	CYP3A4 inhibitor
FHW	None	Yes	None	None	None	None	None
HFW	Yes	Yes	None	None	None	None	None
NVP	None	None	Yes	None	None	None	None

	Excretion	
	Total clearence (log mL/min/kg)	Renal OCT2 substrate
FHW	1.079	Yes
HFW	0.911	Yes
NVP	0.013	None

### 3.6 Toxicology prediction

The toxicity prediction results in Table 8 show that all peptides (FHW and HFW) have no mutagenic, tumorigenic, reproductive effects, or irritant properties, and are free from Pan-Assay Interference Compounds (PAINS) patterns and nasty functions were absent in all peptides. However, both peptides are predicted to have potential as hERG-II inhibitors which related to its potential have cardiotoxicity. The relatively high LD50 values for the three compounds indicate low acute toxicity, with LOAELs for FHW and HFW higher than those for NVP, suggesting a wider safety margin for peptide use. In addition, hepatotoxicity prediction results show that NVP has toxic potential to the liver, while FHW and HFW show no such indication.

**TABLE 8:** Toxicity result prediction.

Compound	Toxicity prediction											
	Mutagenic^a^	Tumorigenic^a^	Reproductive effect^a^	Irritant^a^	PAINS and nasty function^a^	AMES^b^	MTD^b^	hERG-1 inhibitor^b^	hERG-II inhibitor ^b^	LD_50_ ^b^	LOAEL^b^	Hepatotoxicity^b^
FHW	None	None	None	None	None	None	0.445	None	Yes	2.454	3.710	None
HFW	None	None	None	None	None	None	0.445	None	Yes	2.414	2.315	None
NVP	None	None	None	None	None	None	−0.167	None	None	2.715	0.962	Yes

^a^ From DataWarrior.

^b^ From pkCSM, pharmacokinetics.

## 4 Discussion

HIV/AIDS continues to represent a major global health challenge, requiring patients to undergo lifelong ART to control disease progression and reduce mortality. While ART has significantly improved survival, the need for sustained treatment poses several long-term challenges, including cumulative side effects, nonadherence regiment, and a decline in overall quality of life. Improving patient outcomes remains a critical goal with an urgent need to identify alternative or complementary therapeutic strategies that are both effective and better tolerated. In this context, peptide-based therapeutics have emerged as a promising class of agents, offering high specificity, favorable safety profiles, and the potential to overcome drug resistance. However, the traditional process of drug discovery is time-consuming and resource-intensive. To accelerate the identification of promising therapeutic candidates, the integration of computational approaches, for instance, computer-aided drug design, offers a strategic solution to streamline early-phase screening and optimization.

Our study employed an integrated computational approach to identify potential tripeptide inhibitors targeting HIV-1-RT, a key enzyme in viral replication. From a virtual screening of 2,197 tripeptides, three candidates—FHW, HFW, and HHW—demonstrated favorable pharmacological profiles and superior binding affinity compared to Nevirapine (NVP), a widely used NNRTI. Among these, the FHW tripeptide consistently ranked highest across multiple assessment criteria, including docking affinity, binding energy estimates, and predicted safety (Sander et al., 2015; Dalafave, 2010; Morris et al., 2009; Sarthak Pa). The selection of polar amino acids in the construction of tripeptides is based on the ability of polar residues to enhance binding affinity to the target protein (Kudaibergenova et al., 2020; Zhou et al., 2015). Additionally, various antiviral drugs, particularly NNRTIs for HIV-1, tend to possess polar and aromatic functional groups (Mullan et al., 2019).

The integration of IFP-based and chemical property-based ML models significantly improved prediction accuracy and facilitated efficient candidate selection. The QSAR-ML approach showed high predictive performance (IC_50_ *R* ^2^ = 0.955, LELP *R* ^2^ = 0.994), demonstrating its reliability for early-stage screening (Hopkins et al., 2014). Moreover, the binary toxicity evaluation confirmed the non-mutagenic, non-tumorigenic, and non-irritant nature of the selected peptides, a critical aspect for further development.

The superior performance of AdaBoost, Random Forest, and Neural Network models in the first layer suggests that interaction fingerprint-derived features encapsulate rich and discriminative information relevant to ligand-target interactions. The high AUC and MCC scores observed for these models underscore their capacity to discern intricate bioactivity patterns, even in scenarios where class imbalance is a possibility. The persistent underperformance of Naïve Bayes, kNN, and Decision Tree models signifies their inadequacy in high-dimensional or non-linearly separable feature spaces, thereby corroborating the efficacy of ensemble-based approaches for interaction-driven bioactivity modeling.

In the second-layer classification, although the overall performance exhibited a slight decline in comparison to the first layer, Random Forest and AdaBoost once again demonstrated their status as the most robust models. This finding indicates that ensemble methods maintain their efficacy even when applied to a reduced and more specific subset of the data. It is important to note that the second-layer model was not applied across the entire dataset, but rather only on compounds classified as active in the first layer. This underscores its role as a refinement mechanism rather than a direct comparator. This design mirrors real-world virtual screening workflows, where an initial broad filter is followed by a more detailed and chemically focused classification step (Lundberg et al., 2017; Roy et al., 2015; Sahigara et al., 2012; Gramatica, 2007).

The observed decline in performance metrics, particularly in Recall and MCC, can be attributed to several factors. Firstly, there was a reduction in data diversity and sample size. Secondly, the decision boundaries required to distinguish within the active class were more nuanced. However, the consistent performance of Random Forest and AdaBoost indicates that chemical descriptors, despite their reduced discriminatory power compared to interaction fingerprints, nevertheless provide valuable information when utilized in a targeted downstream context.

Docking analyses indicated that the selected peptides bind within the NNRTI allosteric pocket of HIV-1 RT, engaging key residues such as Leu100, Val106, and Tyr188—residues known to be essential for NNRTI activity. The FHW peptide exhibited not only lower binding energy in MM/GBSA and AutoDock4 calculations but also a binding conformation that closely overlapped with that of NVP, suggesting a similar mechanism of inhibition. These findings support the hypothesis that these tripeptides may act as competitive inhibitors by occupying the same allosteric site, thereby disrupting the enzymatic function of RT.

The application of machine learning models—constructed using both interaction fingerprints and physicochemical descriptors—resulted in high predictive performance, as indicated by *R* ^2^ values of 0.955 for IC_50_ and 0.994 for LELP. This integrative QSAR-ML strategy enabled the efficient prioritization of candidate peptides with optimal predicted activity and drug-likeness. Importantly, *in silico* toxicity screening revealed no significant red flags in terms of mutagenicity, tumorigenicity, or irritancy, strengthening the therapeutic potential of the selected candidates (Sander et al., 2015).

Quantum chemical analysis *via* DFT offered additional insight into the electronic characteristics of the lead peptides. FHW exhibited a relatively low HOMO-LUMO gap (4.73 eV) and a high electrophilicity index (13.60), suggesting both favorable chemical reactivity and stability in potential biological environments. These properties are often correlated with improved interaction potential and binding persistence. Furthermore, predicted membrane permeability, assessed using PerMM simulation, indicated that FHW may possess suitable characteristics for passive cellular uptake, although experimental confirmation remains necessary.

The structural behavior of peptide–RT complexes was evaluated through 50-nanosecond MD simulations. The FHW–RT complex demonstrated stable interactions throughout the simulation, with minimal fluctuation in backbone atoms. PCA and DCCM assessments further confirmed the conformational rigidity and favorable dynamic profile of the complex. These data suggest that the FHW peptide maintains a consistent and specific interaction with RT under conditions that approximate the physiological environment.

Quantum mechanics *via* DFT analysis provided deeper insight into the electronic properties of the peptides. The low HOMO-LUMO gap of FHW (4.73 eV) and its high electrophilicity index (13.60) suggest enhanced reactivity and stability, indicating a high likelihood of successful interaction with the RT target. Furthermore, favorable membrane permeability, demonstrated *via* PerMM simulation, supports its potential as an effective orally bioavailable candidate—though this requires further experimental validation.

Molecular dynamics simulations confirmed that the FHW tripeptide forms a uniquely stable complex with HIV-1 reverse transcriptase, significantly outperforming the NVP control and other peptides. The protein’s backbone itself remained stable as represented by RMSD values, and the FHW complex adopted a more consistent and compact fold. This stability was mirrored by the ligand’s own behavior. The FHW peptide quickly settled into a stable pose within the binding pocket, fluctuating similarly to the NVP control. In sharp contrast, both HFW and HHW were far more erratic, suggesting unstable binding. The FHW complex’s stability was further supported by its reduced atomic fluctuations and a consistently lower solvent-accessible surface area, indicating a tight, compact, and well-shielded interaction. Conversely, the high fluctuations seen for HHW across all metrics (RMSD, RMSF, and Rg) suggest it forms a much weaker, more flexible complex that would still allow the protein to change shape.

This exceptional structural stability is driven by a superior binding affinity. MM/GBSA calculations showed the FHW complex had the most favorable binding free energy (ΔG) by a significant margin. This strong attraction is primarily due to substantial van der Waals (ΔG_vdW_) and electrostatic (ΔG_ele_) interactions, which easily overcome the unfavorable solvation energy. Further PCA and DCCM analyses over the 100 ns simulation reinforced these findings, showing that the FHW-RT complex maintains stable, coherent motions-strong evidence of a specific and durable bond under physiological conditions.

Beyond this excellent binding profile, the pharmacokinetic analysis for FHW and HFW also reveals their potential as drug candidates. Both peptides exhibit moderate lipophilicity and membrane permeation characteristics that align with modified Lipinski’s rules for peptide drugs (Lipinski et al., 2001). Interestingly, they appear to be both substrates and inhibitors of P-glycoprotein, a pharmacological paradox that could lead to complex dosing requirements (Sharom, 2008). Finally, their extensive tissue distribution, despite high plasma protein binding, suggests that active transport mechanisms may effectively deliver them to target sites, a phenomenon consistent with modern drug distribution theory (Smith et al., 2010).

The metabolic profiles reveal selective cytochrome P450 interactions that contrast favorably with the broad enzyme inhibition patterns typically associated with drug-drug interactions in HIV therapy (Zanger and Schwab, 2013). The preferential CYP3A4 substrate activity of FHW and the dual CYP2D6/CYP3A4 interaction profile of HFW suggest adherence to established structure-metabolism relationships where minor peptide modifications can significantly alter enzymatic recognition patterns. The high clearance characteristics with active renal transport components support current theories of peptide elimination that emphasize the importance of multiple clearance pathways in maintaining predictable pharmacokinetics, particularly compared to small molecule NNRTIs that rely predominantly on hepatic metabolism (Khodadoust et al., 2021).

Overall, this study provides compelling computational evidence that tripeptides, particularly FHW, could serve as novel RT inhibitors. While *in silico* tools offer significant speed and cost advantages in early drug discovery, the limitations of computational prediction must be addressed through *in vitro* and *in vivo* validation to assess actual bioactivity, pharmacokinetics, and toxicity.

The identification of FHW and related peptides as potential RT inhibitors highlights the growing promise of peptide-based antivirals. Short peptides such as FHW offer several theoretical advantages over conventional small molecules, including enhanced target specificity, reduced off-target effects, and lower toxicity. Moreover, their smaller size relative to longer therapeutic peptides may improve tissue penetration and reduce immunogenicity. The favorable predicted safety and permeability profiles of FHW make it a viable candidate for further development, particularly in populations where resistance to existing NNRTIs is prevalent or adverse effects limit treatment adherence. While oral bioavailability of peptides remains a significant formulation challenge, emerging technologies—such as cyclization, nanoformulation, and carrier-based delivery—offer viable strategies to overcome these limitations. In addition, our studies need to be completed with

## 5 Conclusion

This study presents potential candidates of novel peptide-based inhibitors toward RT-HIV, which offer an alternative to current antiretroviral drugs that often cause adverse side effects and contribute to drug resistance. From a virtual screening of 2,197 non-aliphatic tripeptides, three peptides, namely FHW, HHW, and HFW, emerged as strong candidates for RT-HIV inhibition. These tripeptides exhibit favorable drug-likeness, are predicted to be non-toxic, and demonstrate higher binding affinity to RT-HIV compared to Nevirapine as a widely used commercial drug. DFT analysis showed that the FHW peptide exhibits the most favorable electrophilic nature when compared to the remaining other peptides and Nevirapine. The reliability of these findings was supported by MD simulations and MM/GBSA scoring. These results suggest the potential of FHW, HHW, and HFW to be developed into RT-HIV inhibitors with improved properties over Nevirapine. However, further validation through experimental studies is essential. Future research should focus on evaluating the inhibitory activity of these peptides through *in vitro* and *in vivo* assays.

## Data Availability

The original contributions presented in the study are included in the article/Supplementary Material, further inquiries can be directed to the corresponding author.
