# Supplementary figures and images for: Optimisation of peptides targeting reverse transcriptase HIV-1 using QSAR, machine learning, and computational approaches
Source: Front Pharmacol. 2025 Dec 10;16:1707377. doi: 10.3389/fphar.2025.1707377 (PMC12727953; doi:10.3389/fphar.2025.1707377)

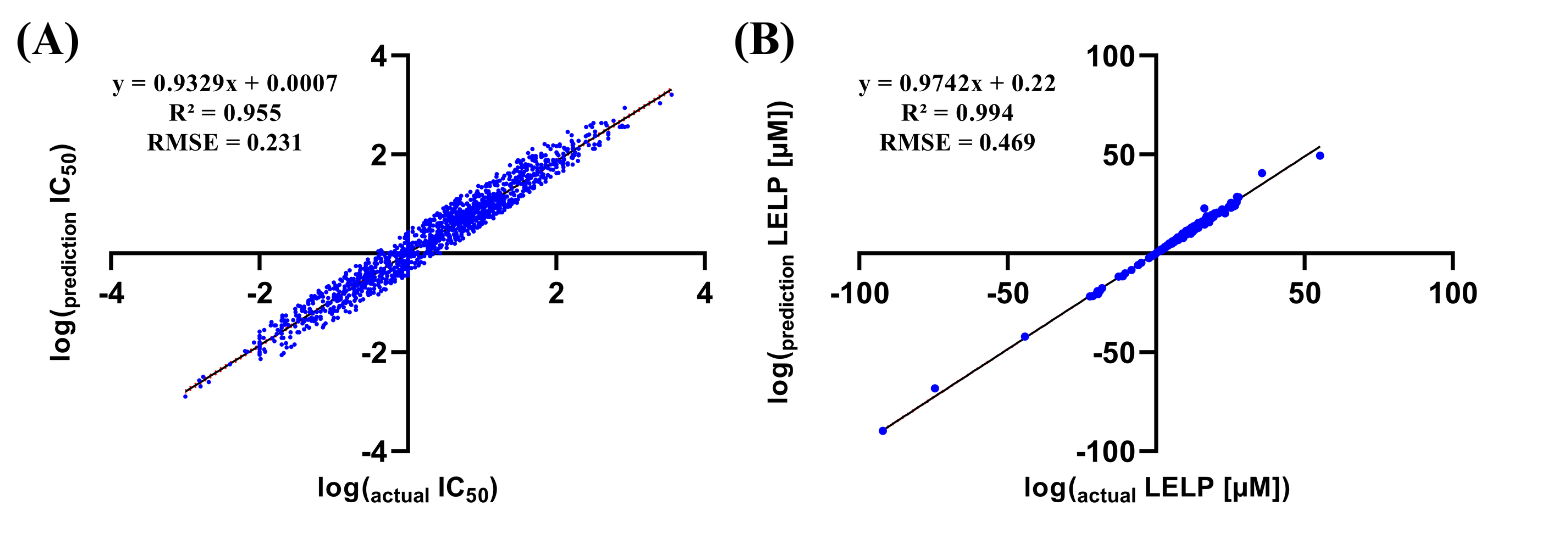

Supplement: Supplementary file 1 [file Image3.tiff]

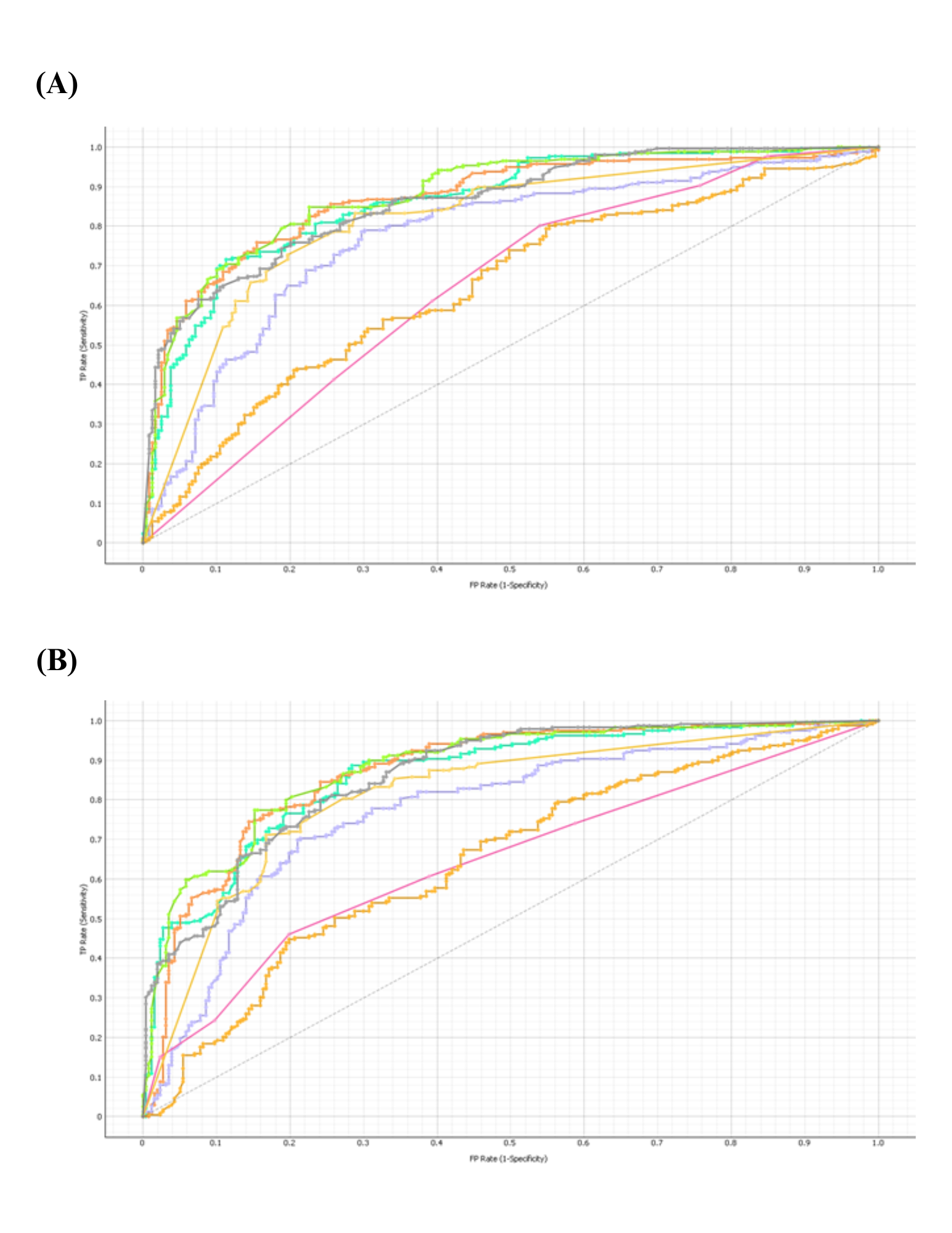

Supplement: Supplementary file 2 [file Image1.tiff]

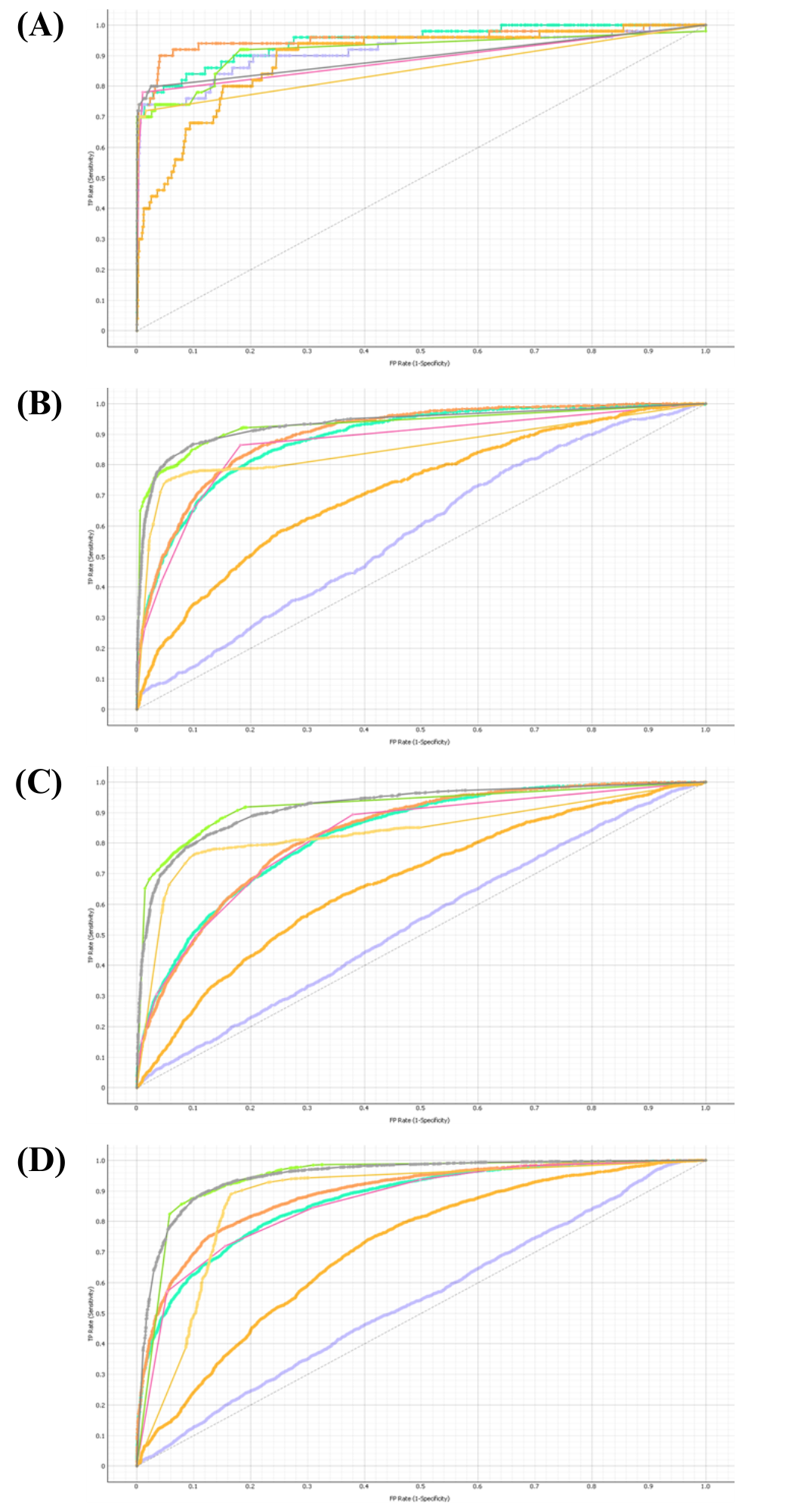

Supplement: Supplementary file 3 [file Image2.tiff]

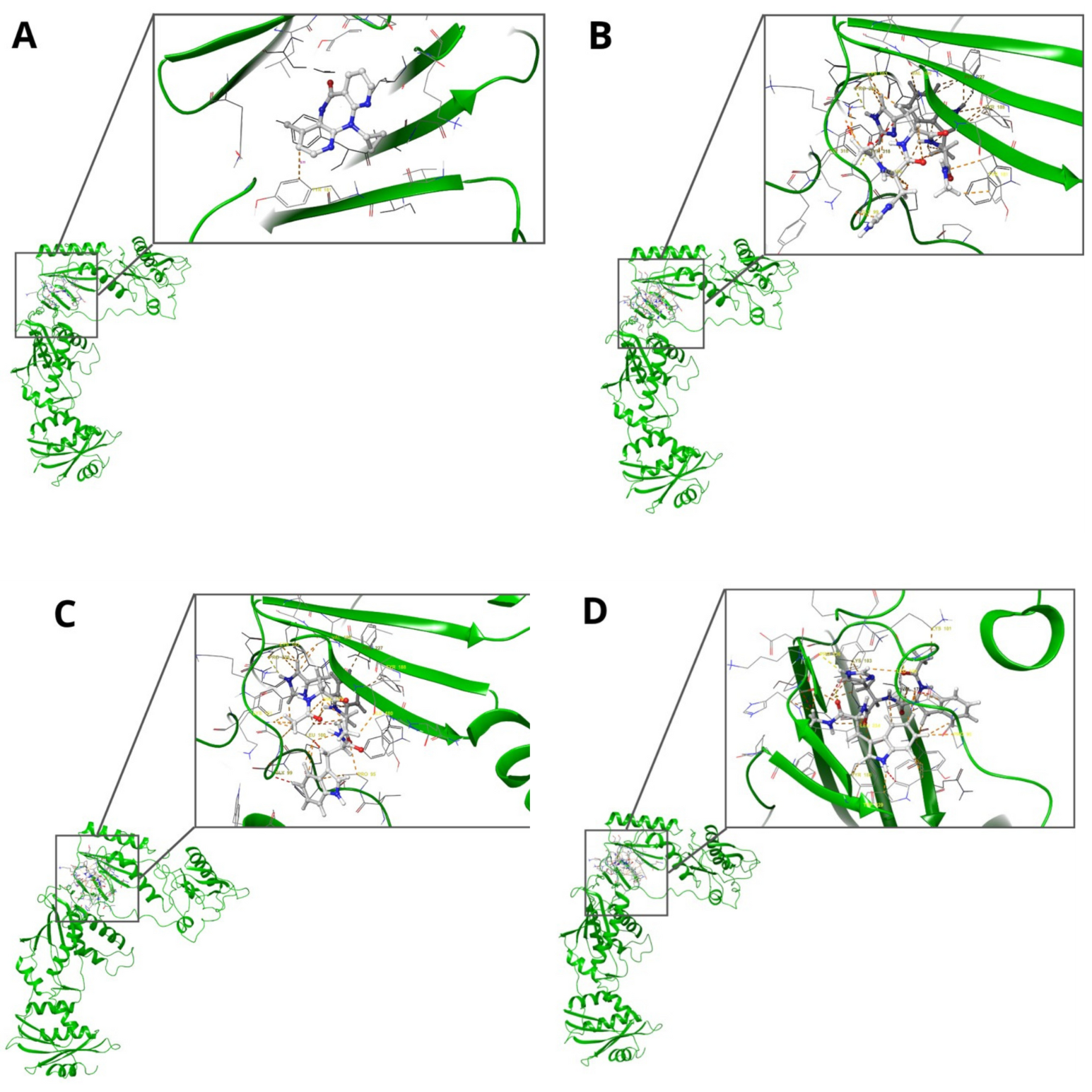

Supplement: Supplementary file 4 [file Image4.tiff]
